# Supplementary material for: Design of Hybrid Quinoline–Chalcone Compounds Against Leishmania amazonensis Based on Computational Techniques: 2D- and 3D-QSAR with Experimental Validation
Source: Pharmaceuticals (Basel). 2025 Oct 17;18(10):1567. doi: 10.3390/ph18101567 (PMC12566680; doi:10.3390/ph18101567)

SUPPLEMENTARY MATERIAL

Design of hybrid quinoline-chalcone compounds against *Leishmania amazonensis* based on computational techniques: 2D- and 3D-QSAR with experimental validation

Marcos Lorca[#1], Gisela C. Muscia[#2*], Jaime Mella[3,4], Luciana Thomaz[5], Jenicer K. Yokoyama-Yasunaka[5], Daniel Moraga[6], Yeray A. Rodríguez-Nuñez[7], Silvia E. Asís[2], Mauro Cortez[5,8], Marco Mellado[9***]**

[1] Facultad de Ciencias de la Vida, Carrera de Química y Farmacia, Universidad Viña del Mar, Viña del Mar 2572002, Chile; marcos.lorca@uvm.cl
[2] Departamento de Ciencias Químicas, Facultad de Farmacia y Bioquímica, Universidad de Buenos Aires, Junín 956, C1113AAB Ciudad Autónoma de Buenos Aires, Argentina; gmuscia@ffyb.uba.ar (G.C.M.), elizabet@ffyb.uba.ar (S.E.A.)
[3] Instituto de Química, Facultad de Ciencias, Universidad de Valparaíso, Av. Gran Bretaña 1111, Valparaíso 2360102, Chile; jaime.mella@uv.cl
[4] Centro de Investigación, Desarrollo e Innovación de Productos Bioactivos (CInBIO), Universidad de Valparaíso, Av. Gran Bretaña 1111, Valparaíso 2360102, Chile.
[5] Institute of Biomedical Sciences. Department of Parasitology, University of São Paulo. Av. Prof. Lineu Prestes 1374 CEP- 05508-000, São Paulo, Brazil; lucithomaz2016@gmail.com (L.T.), jenicerk@usp.br (J.K.Y.Y.), mcortez@usp.br (M.C.)
[6] Laboratorio de Fisiología, Departamento de Ciencias Biomédicas, Facultad de Medicina, Universidad de Tarapacá, Arica, 1000000, Chile; dmoraga@academicos.uta.cl
[7] Universidad Andrés Bello, Facultad de Ciencias Exactas, Departamento de Ciencias Quimicas, Laboratorio de Síntesis y Reactividad de Compuestos Orgánicos, Santiago 8370146; yeray.rodriguez@unab.cl
[8] Escuela de Tecnología Médica, Facultad de Ciencias, Pontificia Universidad Católica de Valparaíso, Valparaíso 2373223, Chile. mauro.cortez@pucv.cl
[9] Centro de Investigación en Ingeniería de Materiales, Universidad Central de Chile, Santiago 8330507, Chile; marco.mellado@ucentral.cl
[#] Authors contributed equally to this study
* Corresponding author: Dr. Gisela Celeste Muscia, Departamento de Ciencias Químicas, Facultad de Farmacia y Bioquímica, Universidad de Buenos Aires, mail: gmuscia@ffyb.uba.ar; Tel.: +54-11-5287-4321.
** Corresponding author: Mauro Cortez, Institute of Biomedical Sciences. Department of Parasitology, Universidad de São Paulo, São Paulo, Brazil; mail: mcortez@usp.br, Tel.: +55-11-3091-7328
*** Corresponding author: Dr. Marco Mellado, Centro de Investigación en Ingeniería de Materiales, Universidad Central de Chile, Santiago 8330507, Chile; marco.mellado@ucentral.cl; Tel.: +56-2-2582-6567

Table of content

1. Theoretical Models

Figure S1: The superimposed structures of all compounds used in the CoMSIA-SA models using the chalcone core. Page 3
Figure S2: Histogram of frequency distribution data for the computational models. Page 4
Table S1: Chemical structure of dataset used to develop the 3D- and 2D-QSAR models. Pages 5-7
Table S2: Field combination of CoMSIA models of Leishmania amazonensis inhibitors Page 8
Table S3: Experimental and predicted pIC50 and residual values for analyzed compounds according to CoMSIA-SA. Page 9
Table S4: *Y*-randomization test for CoMSIA-SA model Page 10
Table S5: Summary of external validation parameters for CoMSIA-SA. Page 11
Table S6: 2D-QSAR results based on electronic descriptors. Page 12

2. *L. amazonensis* inhibition growth

Figure S3: Dose-response graph of the active compounds Page 13

3. Chemistry

^{1}H-NMR, ^{13}C-NMR, and IR spectra of all synthetic compounds Pages 14-39

1. Theoretical Models

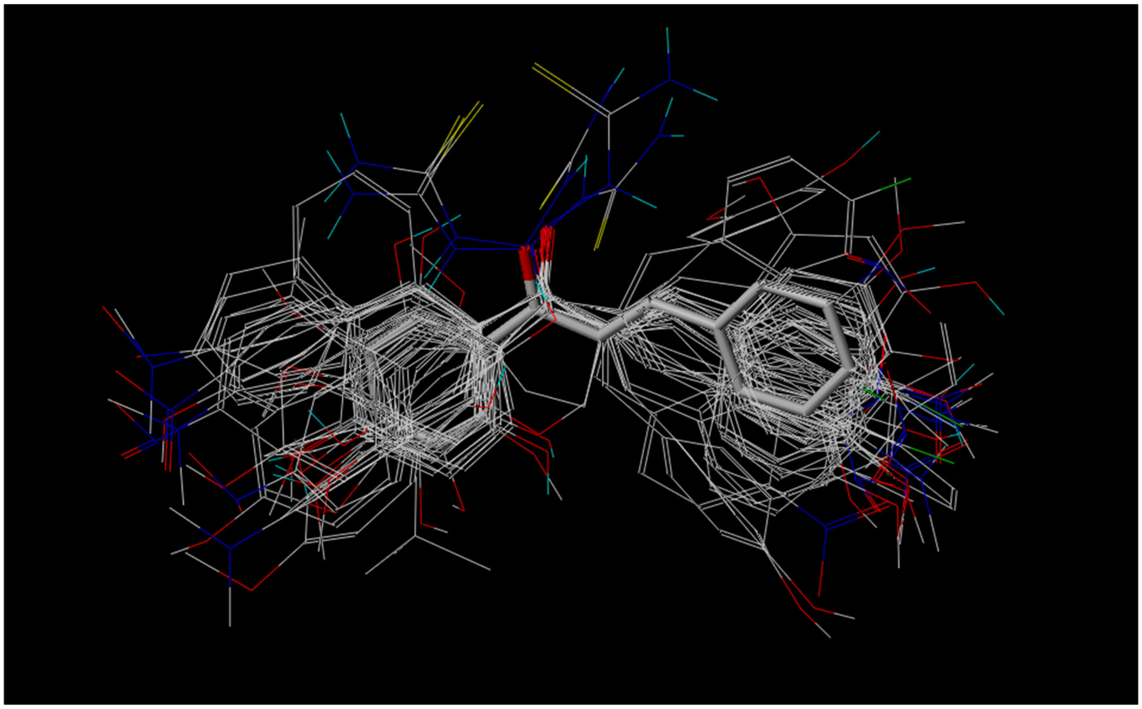

Figure S1: The superimposed structures of all compounds used in the CoMSIA-SA models using the chalcone core.

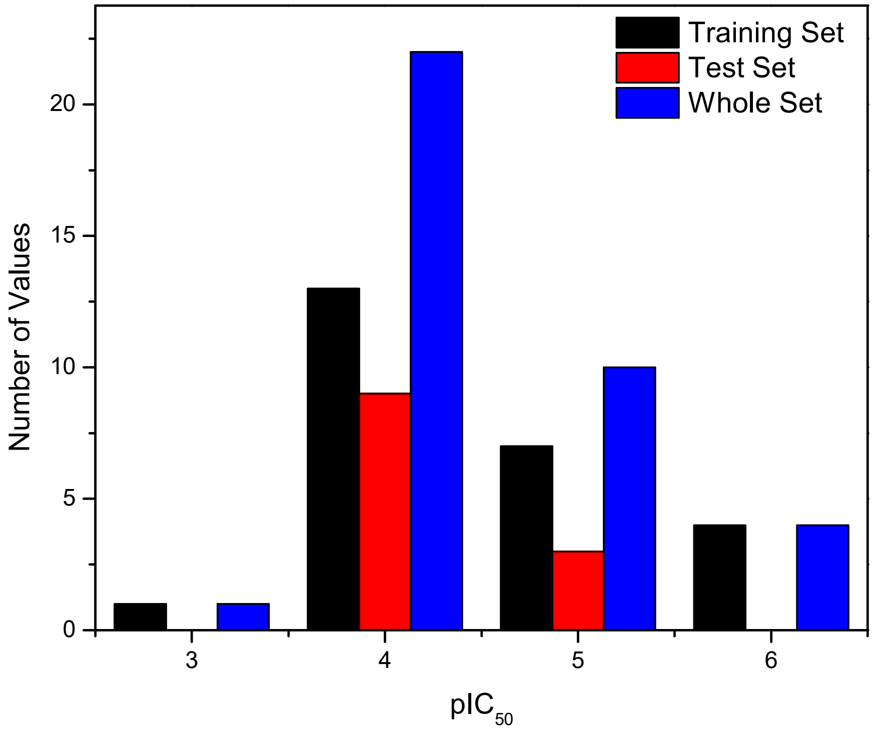

Figure S2: Histogram of frequency distribution data for the computational models.

Table S1: Chemical structure of dataset used to develop the 3D- and 2D-QSAR models.

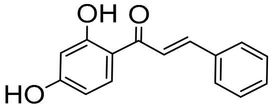<br>001<br>IC_{50}= 0.4x10^{-6} M<br>pIC_{50}= 6.3979 M<br>[1]	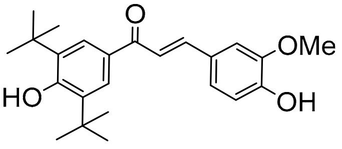<br>002<br>IC_{50}= 6.9x10^{-6} M<br>pIC_{50}= 5.1612 M<br>[2]	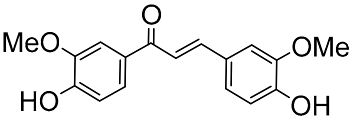<br>003<br>IC_{50}= 20.5x10^{-6} M<br>pIC_{50}= 4.6882 M<br>[2]
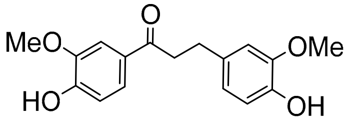<br>004<br>IC_{50}= 16.5x10^{-6} M<br>pIC_{50}= 4.7825 M<br>[2]	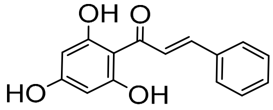<br>005<br>IC_{50}= 3.83x10^{-6} M<br>pIC_{50}= 5.4168 M<br>[3]	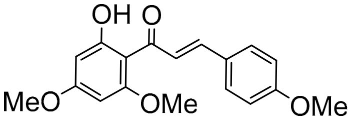<br>006<br>IC_{50}= 7.60x10^{-6} M<br>pIC_{50}= 5.1192 M<br>[3]
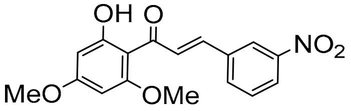<br>007<br>IC_{50}= 0.45x10^{-6} M<br>pIC_{50}= 6.3468 M<br>[3]	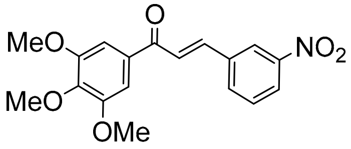<br>008<br>IC_{50}= 0.26x10^{-6} M<br>pIC_{50}= 6.585 M<br>[3]	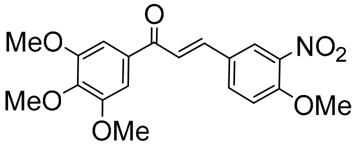<br>009<br>IC_{50}= 2.46x10^{-6} M<br>pIC_{50}= 5.6091 M<br>[3]
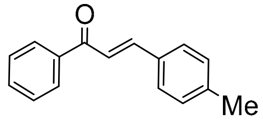<br>010<br>IC_{50}= 14.4x10^{-6} M<br>pIC_{50}= 4.8416 M<br>[3]	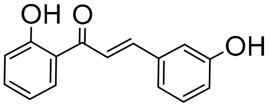<br>011<br>IC_{50}= 0.83x10^{-6} M<br>pIC_{50}= 6.0809 M<br>[3]	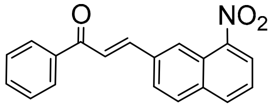<br>012<br>IC_{50}= 1.74x10^{-6} M<br>pIC_{50}= 5.7595 M<br>[3]
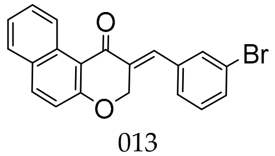<br>013<br>IC_{50}= 4.08x10^{-6} M<br>pIC_{50}= 5.3893 M<br>[3]	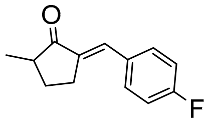<br>014<br>IC_{50}= 8.59x10^{-6} M<br>pIC_{50}= 5.066 M<br>[3]	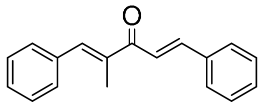<br>015<br>IC_{50}= 15.3x10^{-6} M<br>pIC_{50}= 4.8153 M<br>[4]
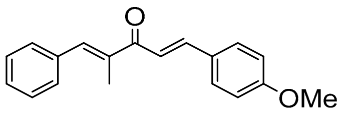<br>016<br>IC_{50}= 20.4x10^{-6} M<br>pIC_{50}= 4.6904 M<br>[4]	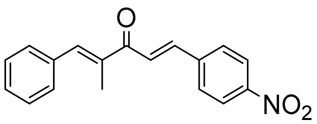<br>017<br>IC_{50}= 11.6x10^{-6} M<br>pIC_{50}= 4.9355 M<br>[4]	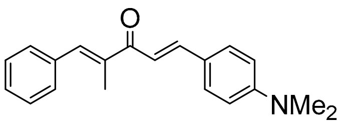<br>018<br>IC_{50}= 20.0x10^{-6} M<br>pIC_{50}= 4.699 M<br>[4]

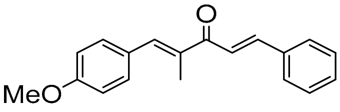<br>019<br>IC$_{50}$= 22.3x10^{-6} M<br>pIC$_{50}$= 4.6517 M<br>[4]	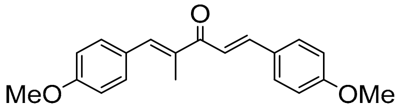<br>020<br>IC$_{50}$= 21.8x10^{-6} M<br>pIC$_{50}$= 4.6615 M<br>[4]
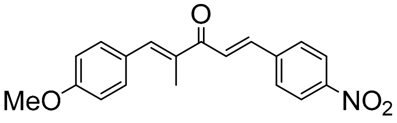<br>021<br>IC$_{50}$= 14.8x10^{-6} M<br>pIC$_{50}$= 4.8297 M<br>[4]	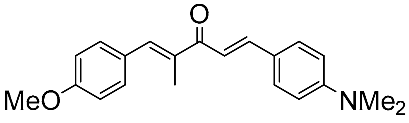<br>022<br>IC$_{50}$= 18.0x10^{-6} M<br>pIC$_{50}$= 4.7447 M<br>[4]
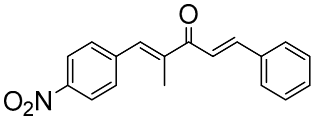<br>023<br>IC$_{50}$= 13.4x10^{-6} M<br>pIC$_{50}$= 4.8729 M<br>[4]	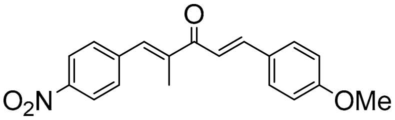<br>024<br>IC$_{50}$= 15.5x10^{-6} M<br>pIC$_{50}$= 4.8097 M<br>[4]
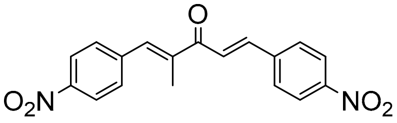<br>025<br>IC$_{50}$= 3.4x10^{-6} M<br>pIC$_{50}$= 5.4685 M<br>[4]	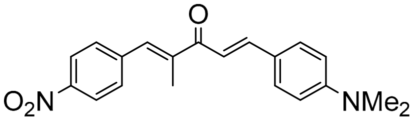<br>026<br>IC$_{50}$= 12.0x10^{-6} M<br>pIC$_{50}$= 4.9208 M<br>[4]
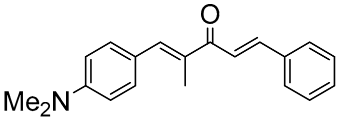<br>027<br>IC$_{50}$= 21.5 x10^{-6} M<br>pIC$_{50}$= 4.6676 M<br>[4]	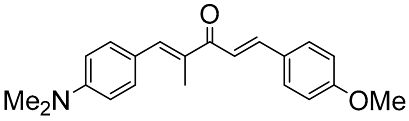<br>028<br>IC$_{50}$= 20.1x10^{-6} M<br>pIC$_{50}$= 4.6968 M<br>[4]
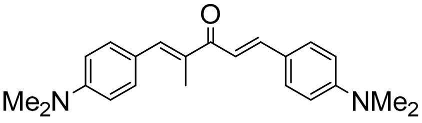<br>029<br>IC$_{50}$= 20.5x10^{-6} M<br>pIC$_{50}$= 4.6882 M<br>[4]	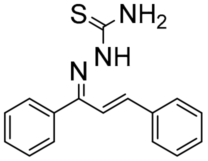<br>030<br>IC$_{50}$= 14.68x10^{-6} M<br>pIC$_{50}$= 4.8333 M<br>[5]

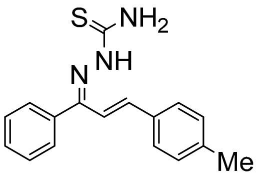<br>031<br>IC_{50}= 14.80x10^{-6} M<br>pIC_{50}= 4.8297 M<br>[5]	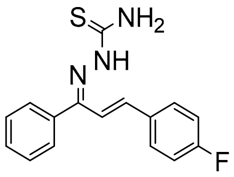<br>032<br>IC_{50}= 13.09x10^{-6} M<br>pIC_{50}= 4.8831 M<br>[5]
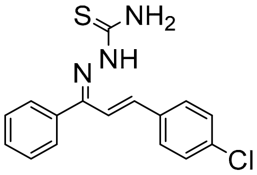<br>033<br>IC_{50}= 5.22x10^{-6} M<br>pIC_{50}= 5.2823 M<br>[5]	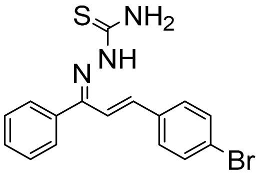<br>034<br>IC_{50}= 12.52x10^{-6} M<br>pIC_{50}= 4.9024 M<br>[5]
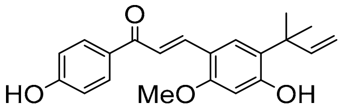<br>035<br>IC_{50}= 3.38x10^{-6} M<br>pIC_{50}= 5.4711 M<br>[6]	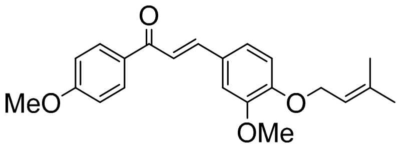<br>036<br>IC_{50}= 67.16x10^{-6} M<br>pIC_{50}= 4.1729 M<br>[6]
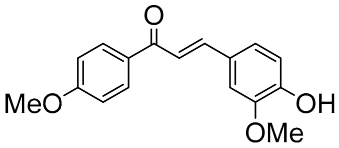<br>037<br>IC_{50}= 287.26x10^{-6} M<br>pIC_{50}= 3.5417 M<br>[6]	

Table S2: Field combination of CoMSIA models of *Leishmania amazonensis* inhibitors

CoMSIA	q^2	N	SEP	SEE	r^2	F	Field Contribution				
							S	E	H	D	A
S	0.749	15	0.567	0.004	1.000	55754.2	1.000				
E	0.451	5	0.576	0.070	0.992	460.3		1.000			
H	0.384	3	0.581	0.166	0.950	132.1			1.000		
D	-0.165	1	0.763	0.576	0.336	11.6				1.000	
A	-0.006	1	0.709	0.546	0.403	15.5					1.000
SE	0.699	11	0.516	0.001	1.000	549047	0.477	0.523			
SEH	0.634	10	0.548	0.001	1.000	1280000	0.327	0.374	0.298		
SEHD	0.539	6	0.542	0.034	0.998	1634.5	0.276	0.274	0.216	0.234	
SEHA	0.586	11	0.605	0.001	1.000	2040000	0.298	0.336	0.264		0.102
SED	0.576	7	0.535	0.023	0.999	3064.3	0.362	0.350		0.283	
SEA	0.633	6	0.484	0.027	0.999	2547.8	0.419	0.448			0.133
SEDA	0.550	7	0.551	0.027	0.999	2276.4	0.344	0.333		0.249	0.074
SH	0.608	2	0.453	0.230	0.899	98	0.538		0.462		
SD	0.629	6	0.486	0.101	0.984	185	0.613			0.387	
SA	0.664	2	0.421	0.167	0.915	129.8	0.775				0.225
SHD	0.540	4	0.514	0.112	0.978	222.8	0.402		0.314	0.285	
SHA	0.582	3	0.478	0.153	0.957	156.8	0.469		0.394		0.137
SDA	0.588	6	0.513	0.112	0.980	149.6	0.570			0.333	0.097
SHDA	0.507	4	0.532	0.114	0.977	216.5	0.374		0.293	0.248	0.085
EH	0.492	11	0.670	0.001	1.000	1820000		0.543	0.457		
ED	0.275	6	0.680	0.049	0.996	785.4		0.612		0.388	
EA	0.396	5	0.604	0.057	0.995	691.6		0.757			0.243
EHD	0.355	6	0.641	0.036	0.998	1451.3		0.394	0.331	0.275	
EHA	0.439	14	0.803	0.000	1.000	6730000		0.462	0.390		0.148
EDA	0.268	7	0.703	0.049	0.997	692.7		0.552		0.319	0.129
EHDA	0.337	6	0.651	0.038	0.998	1309.4		0.364	0.305	0.238	0.093
HD	0.221	7	0.726	0.065	0.994	383.5			0.653	0.347	
HA	0.393	3	0.576	0.14.0	0.964	187.8			0.744		0.256
HDA	0.181	7	0.744	0.072	0.992	311.8			0.576	0.292	0.132
DA	-0.082	1	0.735	0.520	0.459	19.5				0.689	0.311
ALL	0.518	6	0.554	0.034	0.998	1686.6	0.262	0.259	0.206	0.207	0.066

q^2 = the square of the LOO cross-validation (CV) coefficient; N = the optimum number of components; SEP = standard error of prediction; SEE is the standard error of estimation of non CV analysis; r^2_{ncv} is the square of the non CV coefficient; F is the F-test value; S. E. H. D and A are the steric. electrostatic. hydrophobic. hydrogen-bond donor. and hydrogen-bond acceptor contributions respectively.

Table S3: Experimental and predicted pIC_{50} and residual values for analyzed compounds according to CoMSIA-SA.

Compound	pIC_{50}		
	Experimental	Calculated	Residual
**1**	6.398	6.306	0.092
**2**	5.161	5.025	0.136
**3**^{t}	4.688	4.632	0.056
**4**	4.783	5.018	-0.235
**5**	5.417	5.329	0.088
**6**	5.119	5.07	0.049
**7**	6.347	6.339	0.008
**8**	6.585	6.418	0.167
**9**	5.609	5.592	0.017
**10**	4.842	5.145	-0.303
**11**	6.081	6.063	0.018
**12**^{t}	5.759	5.033	0.727
**13**	5.389	5.228	0.161
**14**	5.066	4.852	0.214
**15**	4.815	4.876	-0.061
**16**^{t}	4.69	4.661	0.029
**17**	4.936	4.784	0.152
**18**^{t}	4.699	4.687	0.012
**19**	4.652	4.618	0.034
**20**^{t}	4.662	4.682	-0.021
**21**^{t}	4.83	4.839	-0.009
**22**	4.745	4.85	-0.105
**23**	4.873	5.054	-0.181
**24**	4.81	4.936	-0.126
**25**^{t}	5.469	4.827	0.642
**26**^{t}	4.921	4.668	0.253
**27**	4.668	4.561	0.107
**28**^{t}	4.697	4.775	-0.078
**29**	4.688	4.438	0.25
**30**	4.833	4.475	0.358
**31**^{t}	4.83	5.215	-0.385
**32**	4.883	5.191	-0.308
**33**^{t}	5.282	5.311	-0.029
**34**^{t}	4.902	4.925	-0.023
**35**	5.471	5.741	-0.27
**36**	4.173	3.993	0.18
**37**	3.542	3.986	-0.444

^{t}= Test set

Table S4: *Y*-randomization test for CoMSIA-SA model

Iteration	q^2	N	r^2_{ncv}	Iteration	q^2	N	r^2_{ncv}
**Random 1**	-0.531	1	0.259	Random 6	-0.301	2	0.736
**Random 2**	-0.821	1	0.369	Random 7	-0.118	4	0.909
**Random 3**	0.068	2	0.681	Random 8	-0.458	1	0.353
**Random 4**	-0.128	3	0.813	Random 9	-0.806	1	0.393
**Random 5**	-0.887	1	0.416	Random 10	-0.32	1	0.34

Table S5. Summary of external validation parameters for CoMSIA-SA.

Condition	Parameters	Threshold value	CoMSIA
**1**	q^2	>0.5	0.664
**2**	r_{test}^2	>0.6	0.915
**3a**	r_0^2	Close to value of r_{test}^2	0.967
**3b**	r'^2_0	Close to value of r_{test}^2	0.962
**4a**	k	0.85 < k < 1.15	1.002
**4b**	k'	0.85 < k′ < 1.15	0.998
**5a**	$(r^2 - r_0^2)/r^2$	<0.1	0.051
**5b**	$(r^2 - r'^2_0)/r^2$	<0.1	0.051
**6**	$\lvert r_0^2 - r'^2_0 \rvert$	<0.3	0.005
**7**	r_m^2	>0.5	0.706

q^2 and r^2_{test} are the same parameters as listed in Table S2; r_0^2 and k are the correlation coefficient between the actual and predicted activities for test set and the respective slope of regression; and $r_0'^2$ and k′ are the correlation coefficient between the predicted and actual activities for test set and the respective slope of regression. r^2_m was defined in equation 4.

Table S6: 2D-QSAR results based on electronic descriptors.

Compound	LUMO	LUMO²	ω	ω²	pIC₅₀ (M)		Residual
					Experimental	Calculated	
**1**	-2.195	4.817	4.411	19.456	6.398	5.563	0.835
**2**ᵗ	-1.744	3.042	3.503	12.268	5.161	4.377	0.784
**3**	-1.718	2.953	3.464	11.999	4.688	4.384	0.304
**4**	-1.299	1.688	2.795	7.81	4.783	4.788	-0.005
**5**ᵗ	-2.333	5.442	4.75	22.561	5.417	5.972	-0.555
**6**ᵗ	-1.737	3.019	3.49	12.18	5.119	4.366	0.754
**7**	-2.603	6.776	5.435	29.541	6.347	6.391	-0.044
**8**	-2.682	7.194	5.604	31.405	6.585	6.487	0.098
**9**	-2.355	5.544	4.768	22.735	5.609	6.022	-0.413
**10**	-1.897	3.598	3.808	14.502	4.842	4.762	0.08
**11**	-2.296	5.272	4.647	21.592	6.081	5.87	0.211
**12**ᵗ	-2.785	7.758	5.827	33.957	5.76	6.579	-0.819
**13**	-2.264	5.126	4.582	20.991	5.389	5.788	-0.398
**14**	-1.871	3.501	3.762	14.149	5.066	4.716	0.35
**15**ᵗ	-2.127	4.522	4.258	18.128	4.815	5.338	-0.522
**16**	-1.962	3.851	3.926	15.412	4.69	4.884	-0.194
**17**ᵗ	-2.965	8.794	6.402	40.984	4.936	5.764	-0.828
**18**	-1.752	3.068	3.504	12.278	4.699	4.334	0.365
**19**ᵗ	-2.018	4.073	4.049	16.392	4.652	5.064	-0.412
**20**	-1.858	3.453	3.717	13.813	4.662	4.605	0.057
**21**	-2.885	8.323	6.422	41.245	4.83	4.828	0.002
**22**ᵗ	-1.644	2.704	3.29	10.827	4.745	4.068	0.676
**23**ᵒ	-2.762	7.628	5.729	32.825	4.873	6.706	-1.833
**24**ᵒ	-2.663	7.093	5.622	31.608	4.81	6.304	-1.494
**25**ᵗ	-3.263	10.647	7.043	49.606	5.469	5.471	-0.003
**26**ᵒ	-2.529	6.396	5.498	30.227	4.921	5.88	-0.959
**27**ᵗ	-1.856	3.446	3.735	13.95	4.668	4.691	-0.024
**28**	-1.704	2.904	3.41	11.627	4.697	4.214	0.482
**29**	-1.509	2.278	3.027	9.164	4.688	3.789	0.899
**30**ᵗ	-1.927	3.714	3.868	14.958	4.833	4.835	-0.002
**31**ᵗ	-1.848	3.417	3.701	13.695	4.83	4.595	0.235
**32**ᵗ	-1.965	3.861	3.948	15.588	4.883	4.949	-0.066
**33**ᵗ	-2.104	4.428	4.266	18.199	5.282	5.402	-0.119
**34**	-2.107	4.441	4.274	18.264	4.902	5.406	-0.504
**35**ᵒ	-1.601	2.564	3.235	10.465	5.471	4.147	1.324
**36**ᵗ	-1.837	3.374	3.676	13.514	4.173	4.56	-0.387
**37**ᵗ	-1.78	3.17	3.565	12.708	3.542	4.422	-0.88

ᵗ= test set; ⁰= outlier compound

2. *L. amazonensis* inhibition growth

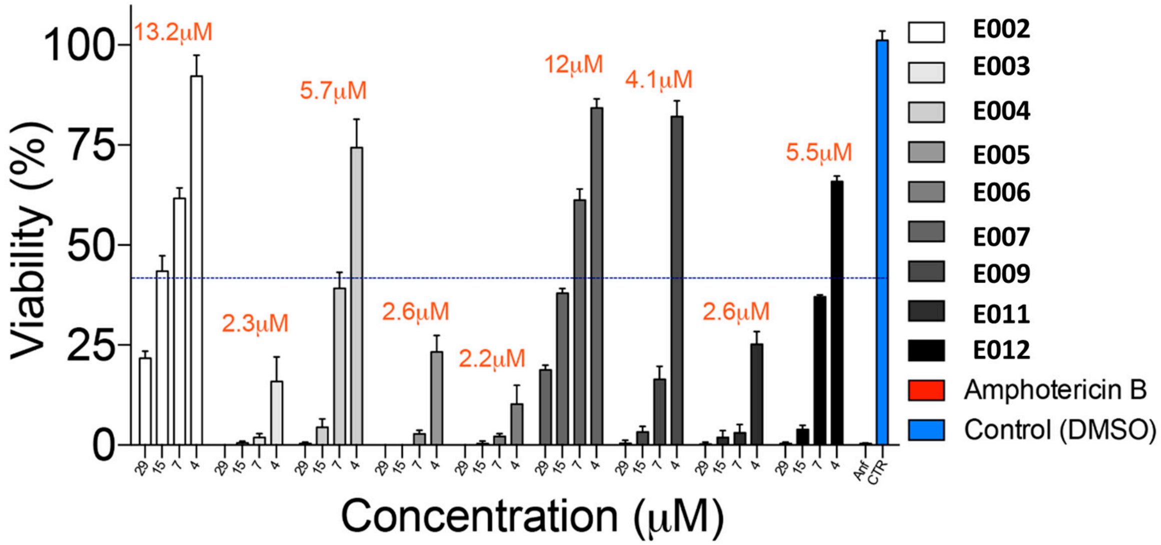

Figure S3: Dose-response graph of the active compounds

3. Chemistry

Spectrum S1: ^{1}H-NMR ($CDCl_3$). 1-(6-chloro-4-(2-fluorophenyl)-2-methylquinolin-3-yl)ethan-1-one **(III)**

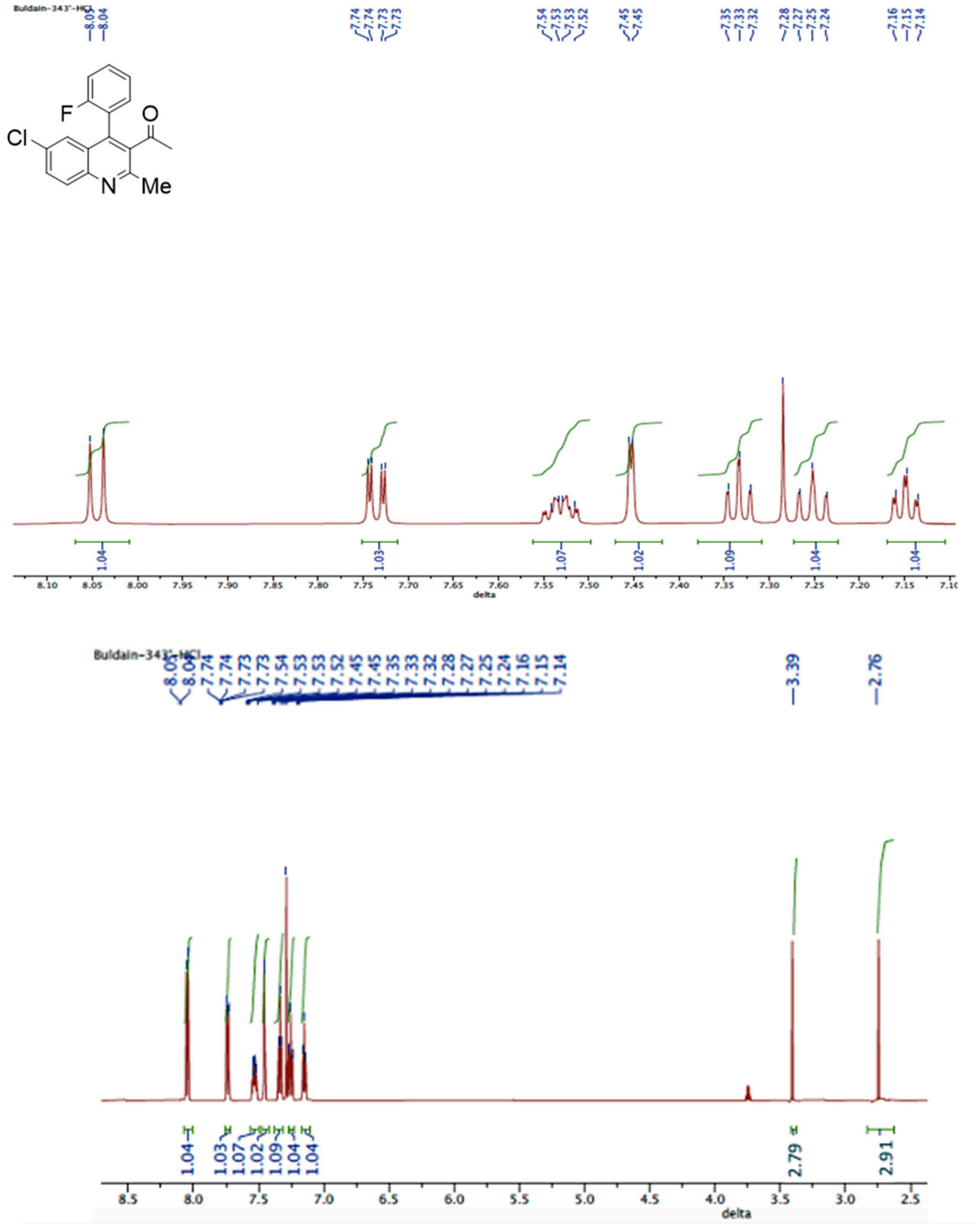

Spectrum S2: ^{13}C-NMR ($CDCl_3$). 1-(6-chloro-4-(2-fluorophenyl)-2-methylquinolin-3-yl)ethan-1-one **(III)**

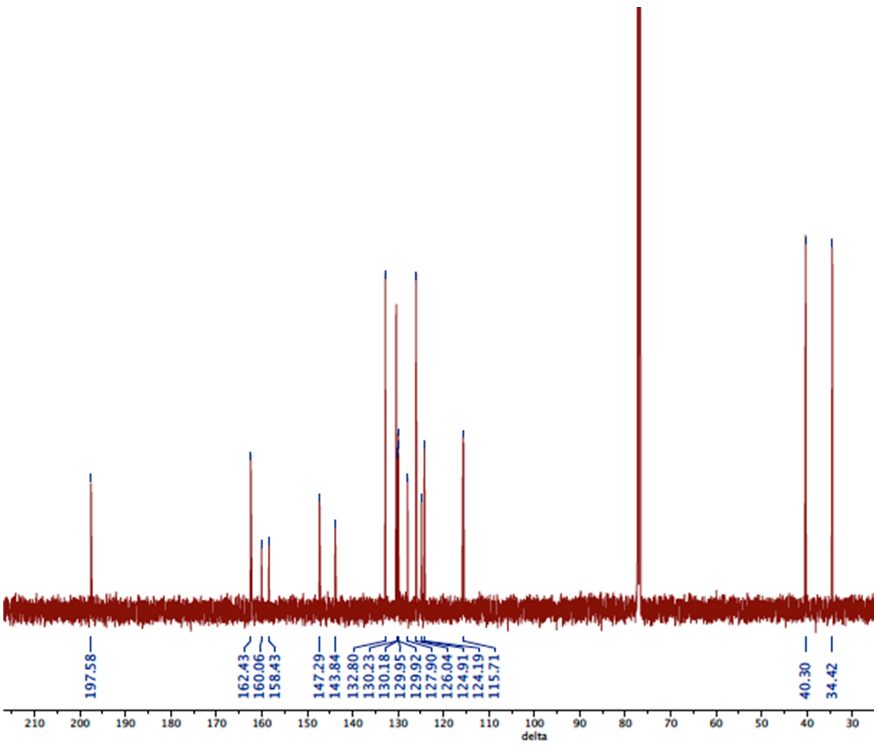

Spectrum S3: IR (KBr). 1-(6-chloro-4-(2-fluorophenyl)-2-methylquinolin-3-yl)ethan-1-one **(III)**

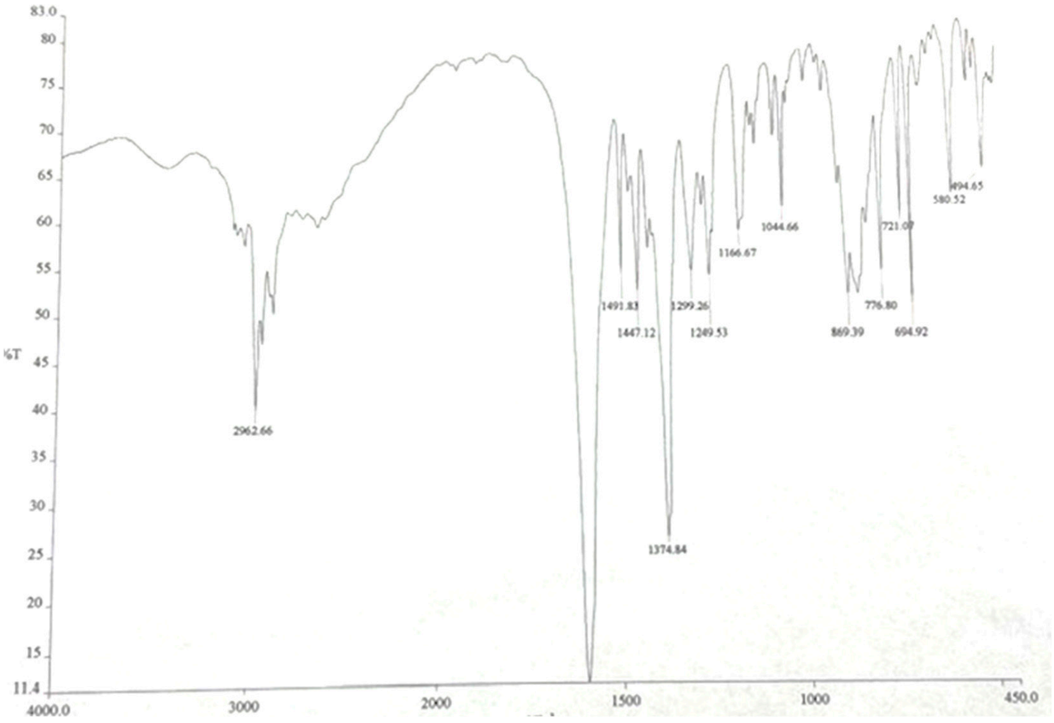

Spectrum S4: ^{1}H-NMR (DMSO-d_6). (*E*)-1-(6-chloro-4-(2-fluorophenyl)-2-methylquinolin-3-yl)-3-(3,4-dimetoxyphenyl)prop-2-en-1-one **(E001)**

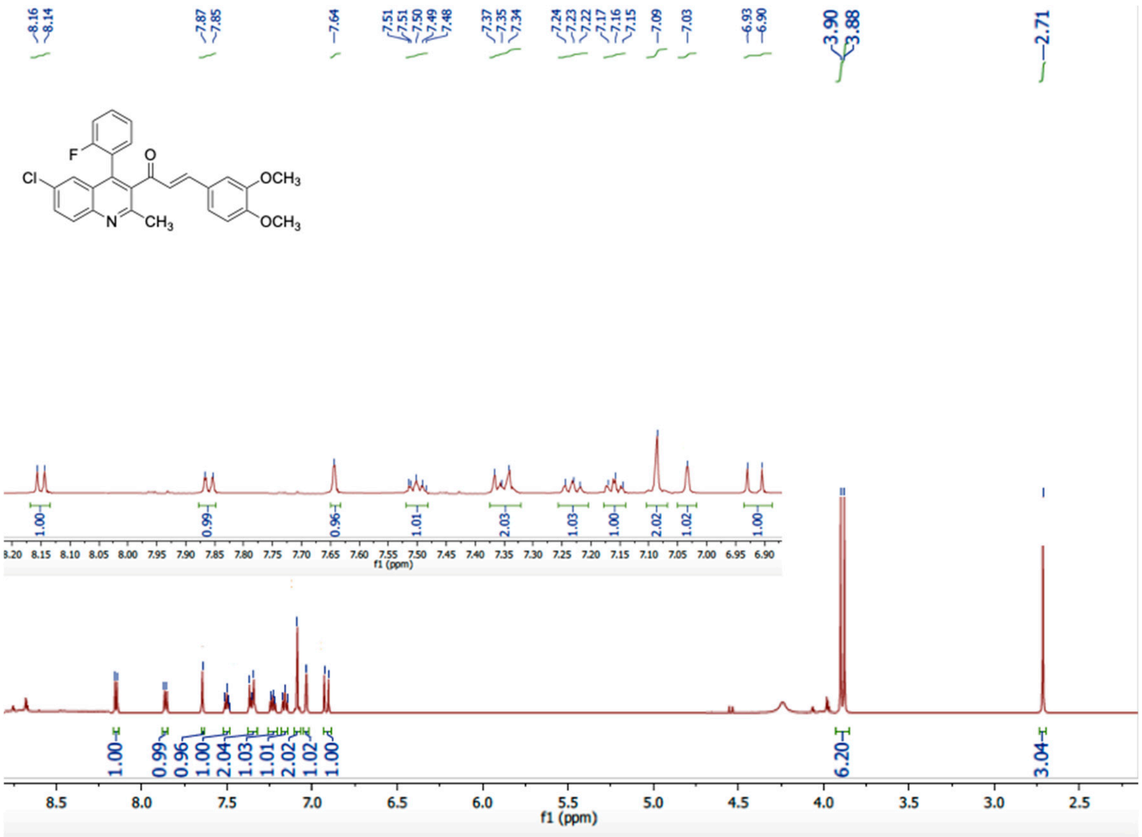

Spectrum S5: ^{13}C-NMR (DMSO-d^6). (*E*)-1-(6-chloro-4-(2-fluorophenyl)-2-methylquinolin-3-yl)-3-(3,4-dimetoxyphenyl)prop-2-en-1-one **(E001)**

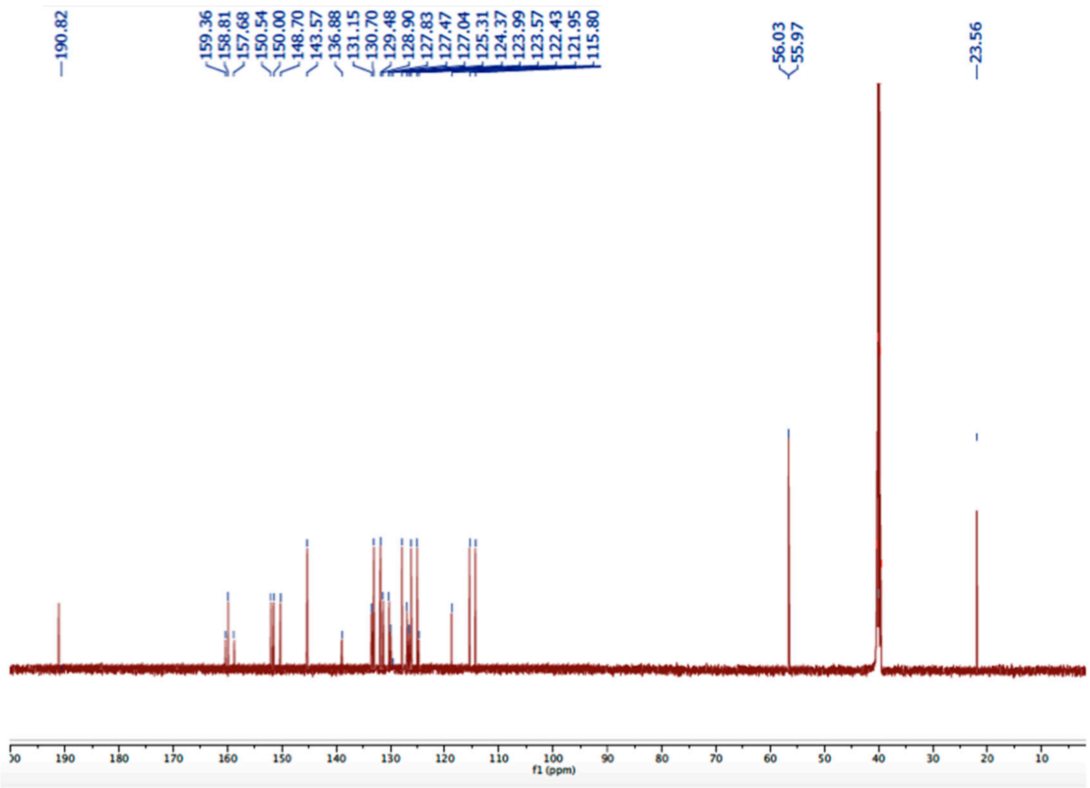

Spectrum S6: IR (KBr). (*E*)-1-(6-chloro-4-(2-fluorophenyl)-2-methylquinolin-3-yl)-3-(3,4-dimetoxyphenyl)prop-2-en-1-one **(E001)**

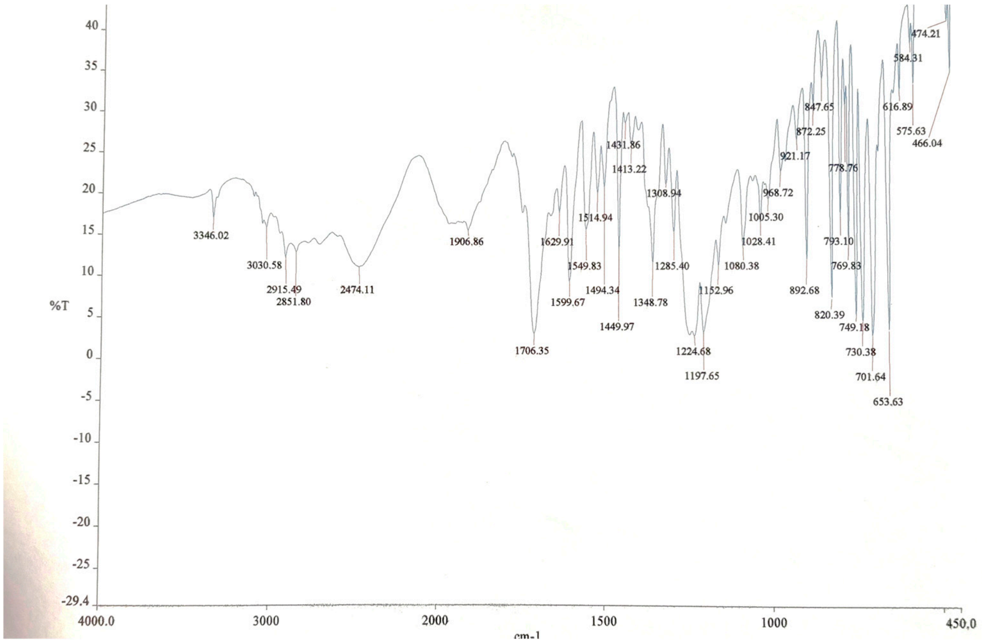

Spectrum S7: ^{1}H-NMR (DMSO-d_6). (*E*)-3-(benzo[d][1,3]dioxol-5-yl)1-(6-chloro-4-(2-fluorophenyl)-2-methylquinolin-3-yl)prop-2-en-1-one **(E002)**

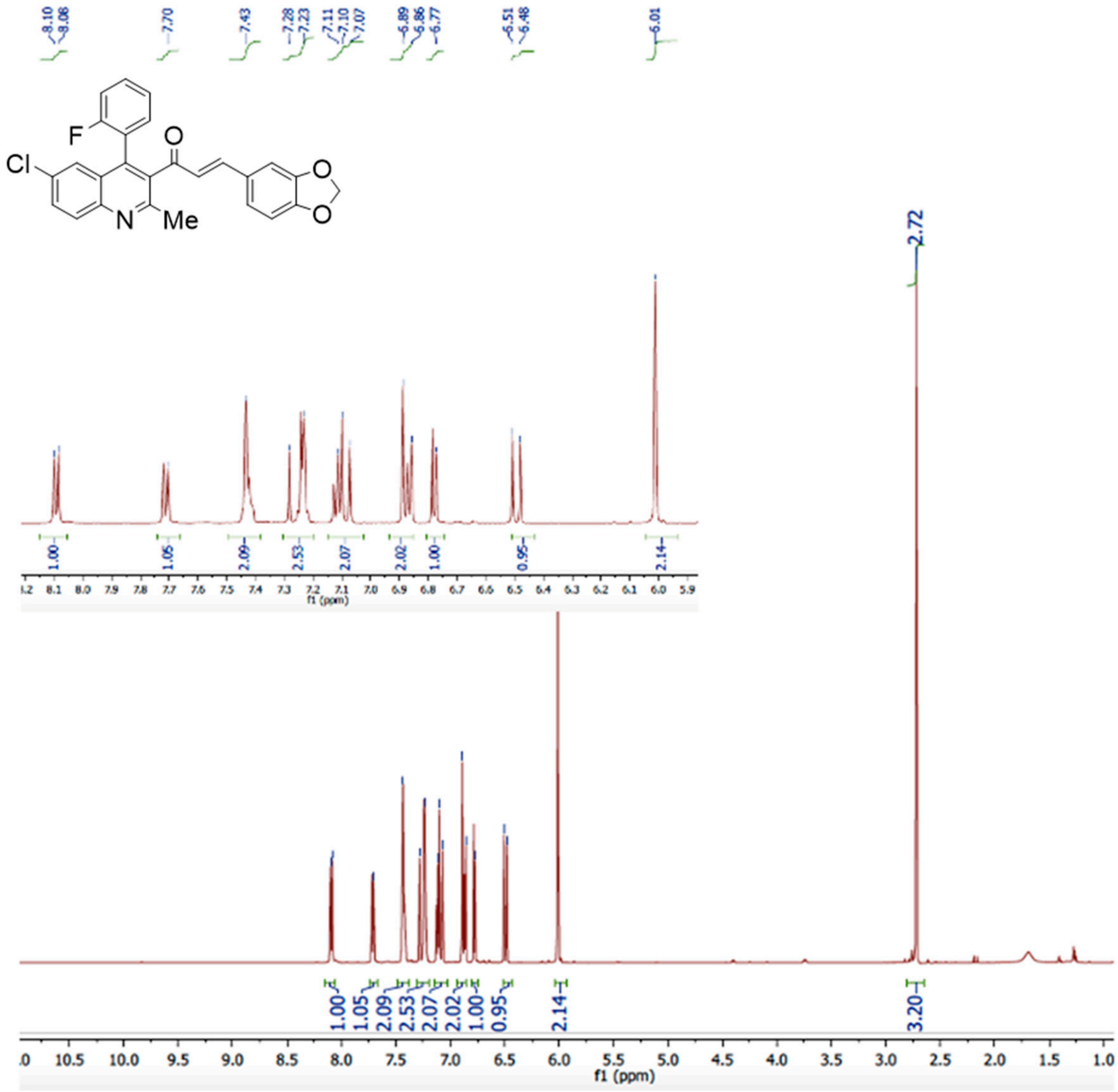

Spectrum S8: ^{13}C-NMR (DMSO-d_6). (*E*)-3-(benzo[d][1,3]dioxol-5-yl)1-(6-chloro-4-(2-fluorophenyl)-2-methylquinolin-3-yl)prop-2-en-1-one **(E002)**

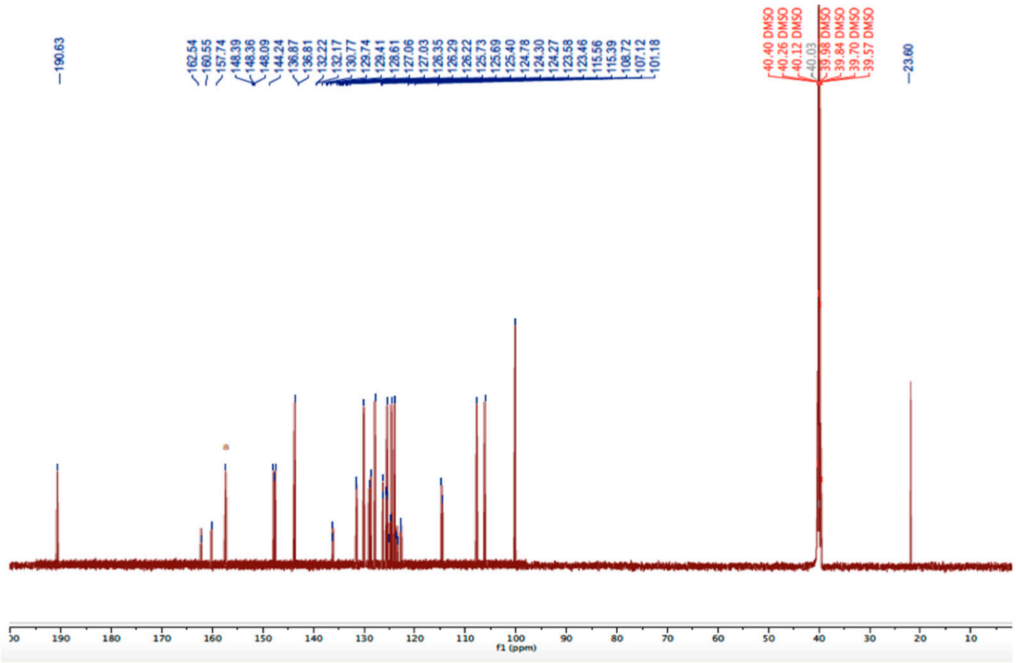

Spectrum S9: IR (KBr). (*E*)-3-(benzo[d][1,3]dioxol-5-yl)1-(6-chloro-4-(2-fluorophenyl)-2-methylquinolin-3-yl)prop-2-en-1-one **(E002)**

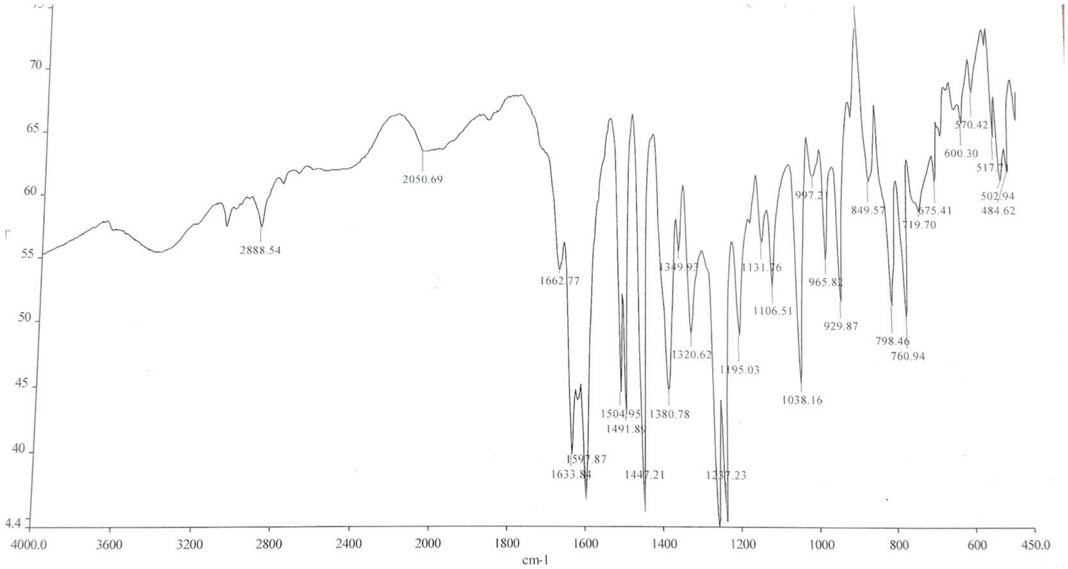

Spectrum S10: ^{1}H-NMR (DMSO-d_6). (*E*)-1-(6-chloro-4-(2-fluorophenyl)-2-methylquinolin-3-yl)-3-(3,4-difluorophenyl)prop-2-en-1-one **(E003)**

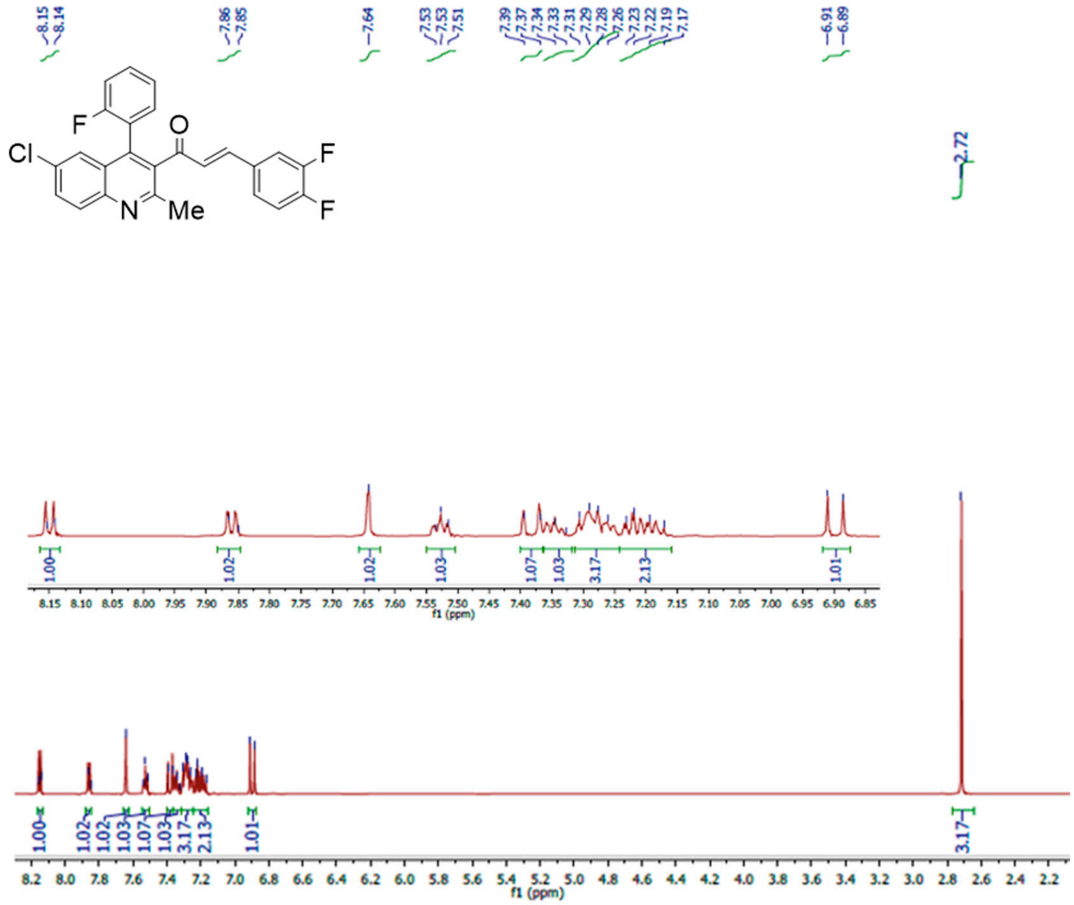

Spectrum S11: ^{13}C-NMR (DMSO-d_6). (*E*)-1-(6-chloro-4-(2-fluorophenyl)-2-methylquinolin-3-yl)-3-(3,4-difluorophenyl)prop-2-en-1-one **(E003)**

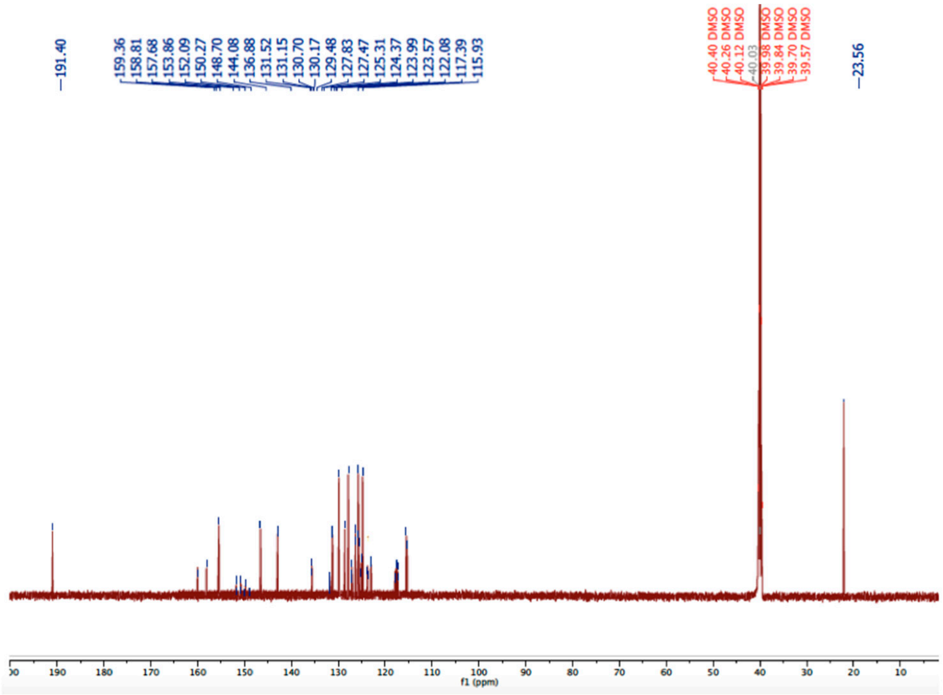

Spectrum S12: IR (KBr). (E)-1-(6-chloro-4-(2-fluorophenyl)-2-methylquinolin-3-yl)-3-(3,4-difluorophenyl)prop-2-en-1-one **(E003)**

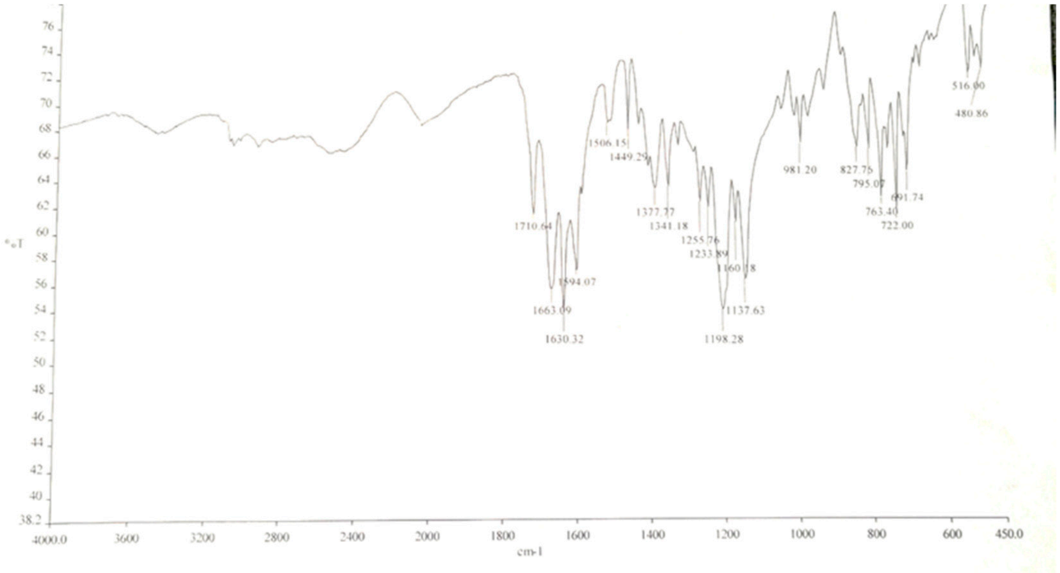

Spectrum S13: ^{1}H-NMR (DMSO-d_6). (*E*)-1-(6-chloro-4-(2-fluorophenyl)-2-methylquinolin-3-yl)-3-(3,4,5-trimetoxyphenyl)prop-2-en-1-one **(E004)**

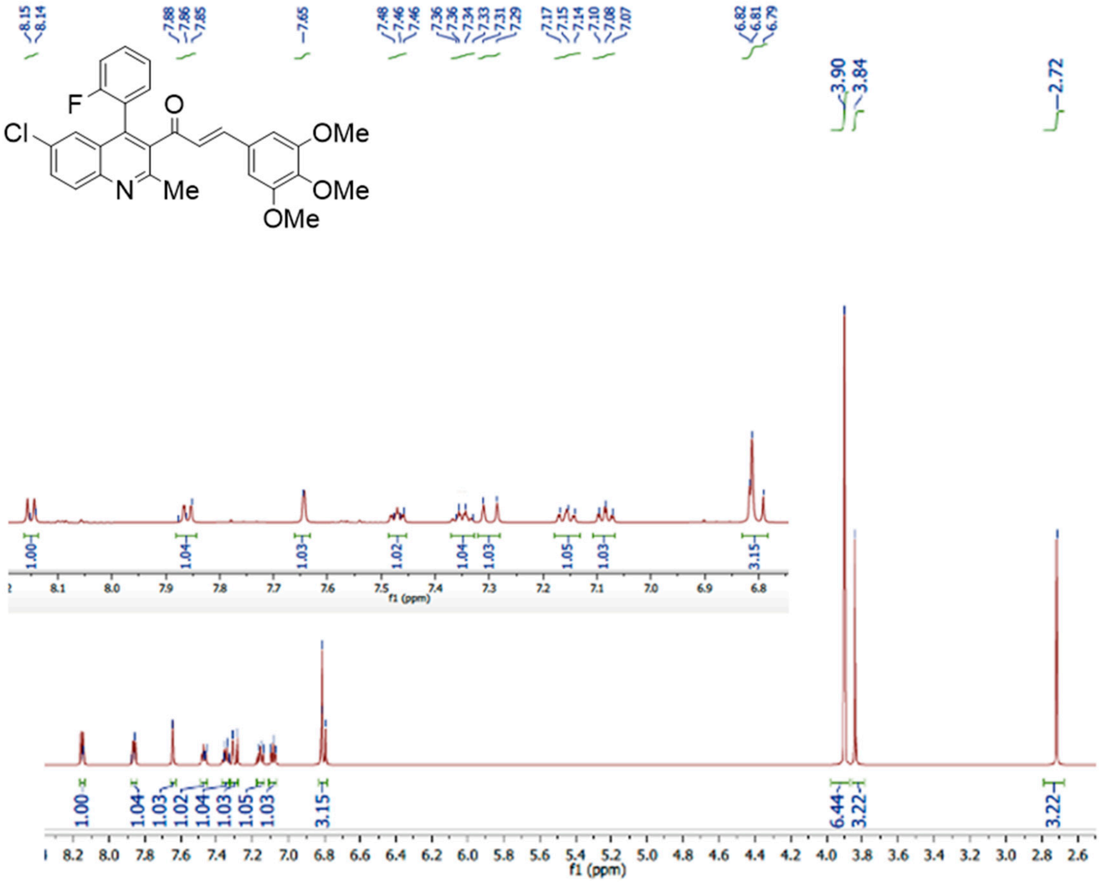

Spectrum S14: ^{13}C-NMR (DMSO-d_6). (*E*)-1-(6-chloro-4-(2-fluorophenyl)-2-methylquinolin-3-yl)-3-(3,4,5-trimetoxyphenyl)prop-2-en-1-one **(E004)**

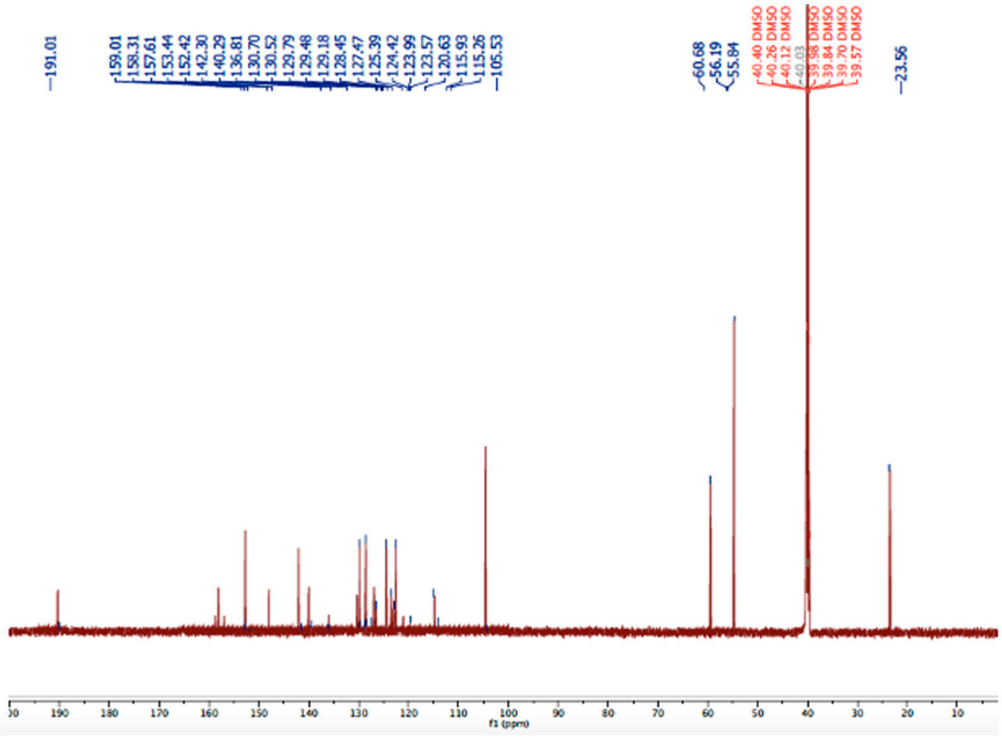

Spectrum S15: IR (KBr). (*E*)-1-(6-chloro-4-(2-fluorophenyl)-2-methylquinolin-3-yl)-3-(3,4,5-trimetoxyphenyl)prop-2-en-1-one **(E004)**

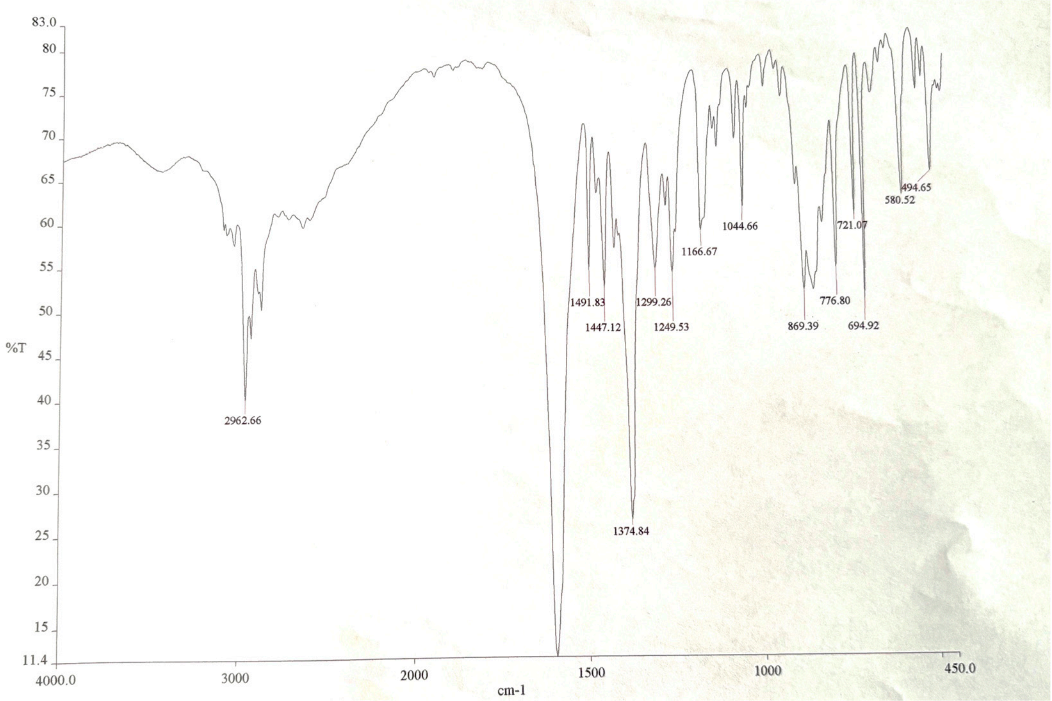

Spectrum S16: ^{1}H-NMR (DMSO-d$_6$). (*E*)-1-(6-chloro-4-(2-fluorophenyl)-2-methylquinolin-3-yl)-3-(4-(trifluoromethyl)phenyl)prop-2-en-1-one **(E005)**

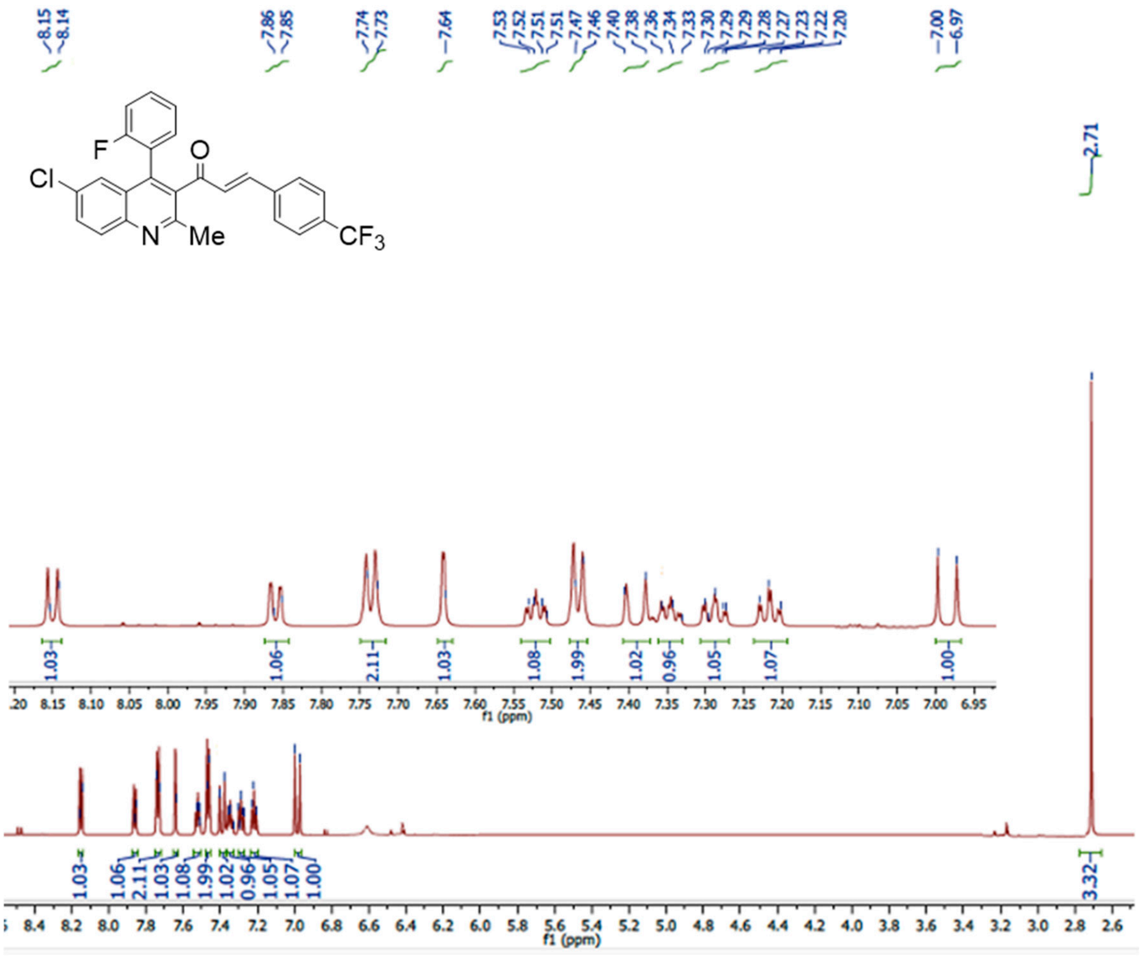

Spectrum S17: ^{13}C-NMR (DMSO-d_6). (*E*)-1-(6-chloro-4-(2-fluorophenyl)-2-methylquinolin-3-yl)-3-(4-(trifluoromethyl)phenyl)prop-2-en-1-one **(E005)**

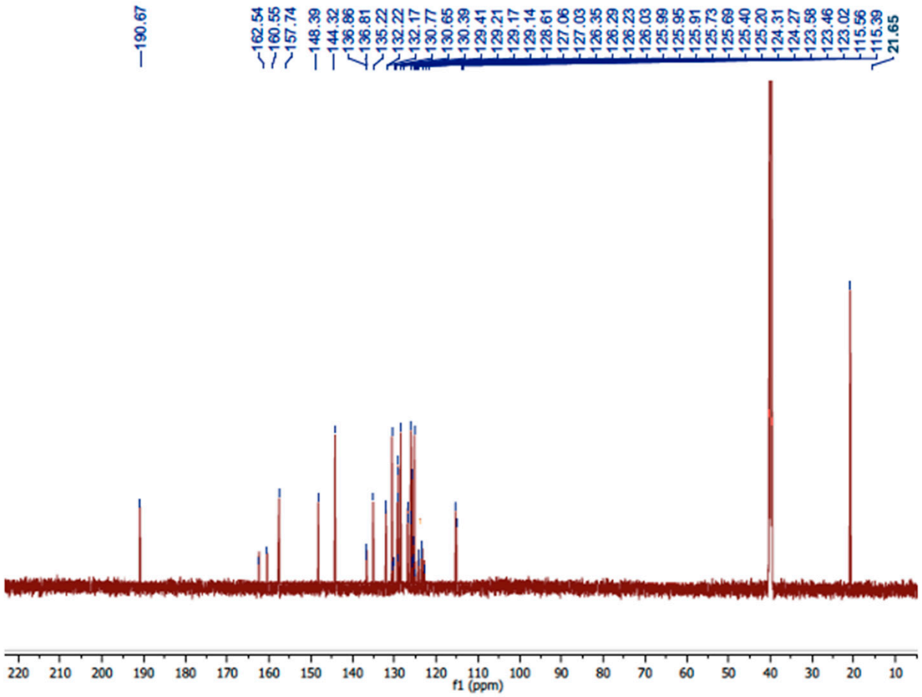

Spectrum S18: IR (KBr). (*E*)-1-(6-chloro-4-(2-fluorophenyl)-2-methylquinolin-3-yl)-3-(4-(trifluoromethyl)phenyl)prop-2-en-1-one **(E005)**

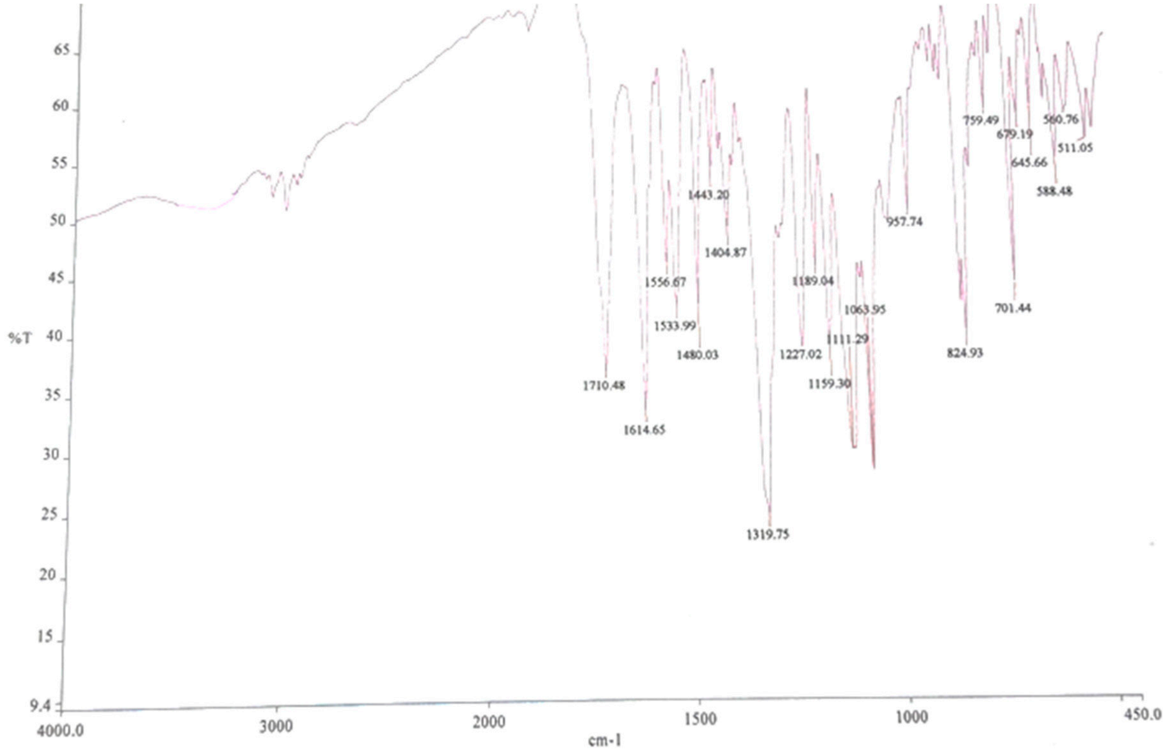

Spectrum S19: ^{1}H-NMR (DMSO-d$_6$). (*E*)-1-(6-chloro-4-(2-fluorophenyl)-2-methylquinolin-3-yl)-3-(3,4-dichlorophenyl)prop-2-en-1-one **(E006)**

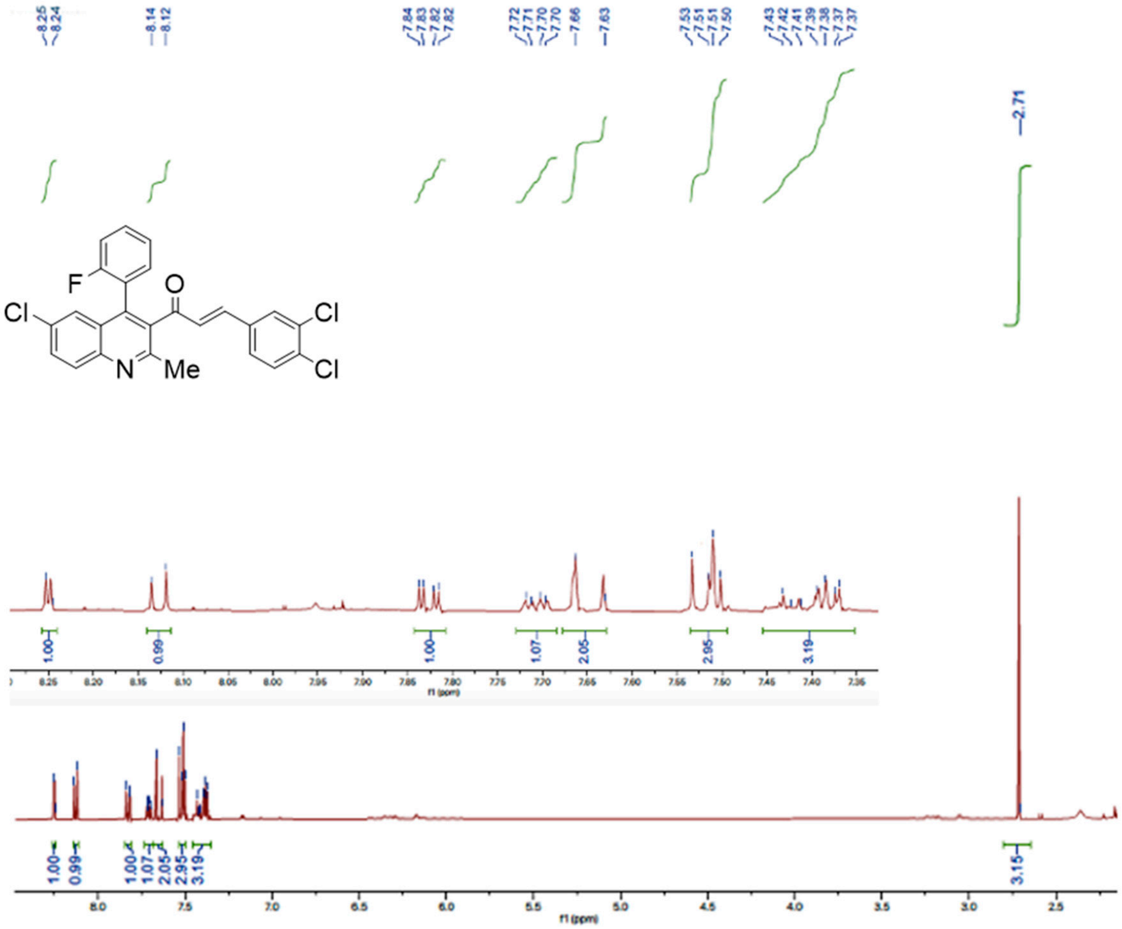

Spectrum S20: ^{13}C-NMR (DMSO-d_6). (*E*)-1-(6-chloro-4-(2-fluorophenyl)-2-methylquinolin-3-yl)-3-(3,4-dichlorophenyl)prop-2-en-1-one **(E006)**

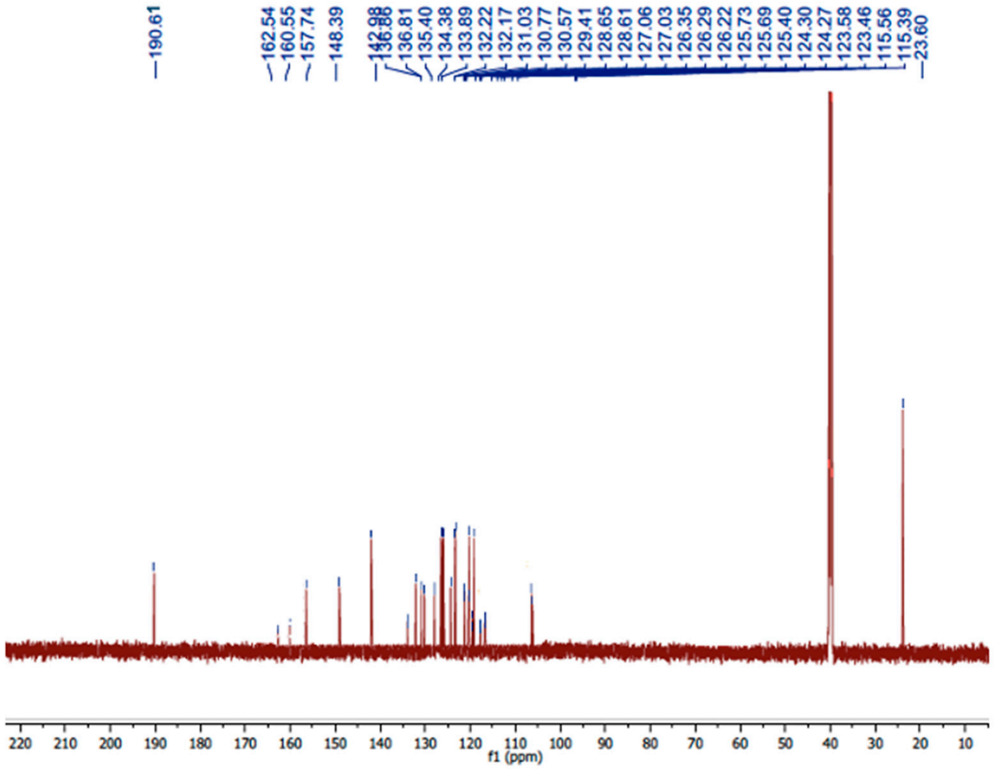

Spectrum S21: IR (KBr). (*E*)-1-(6-chloro-4-(2-fluorophenyl)-2-methylquinolin-3-yl)-3-(3,4-dichlorophenyl)prop-2-en-1-one **(E006)**

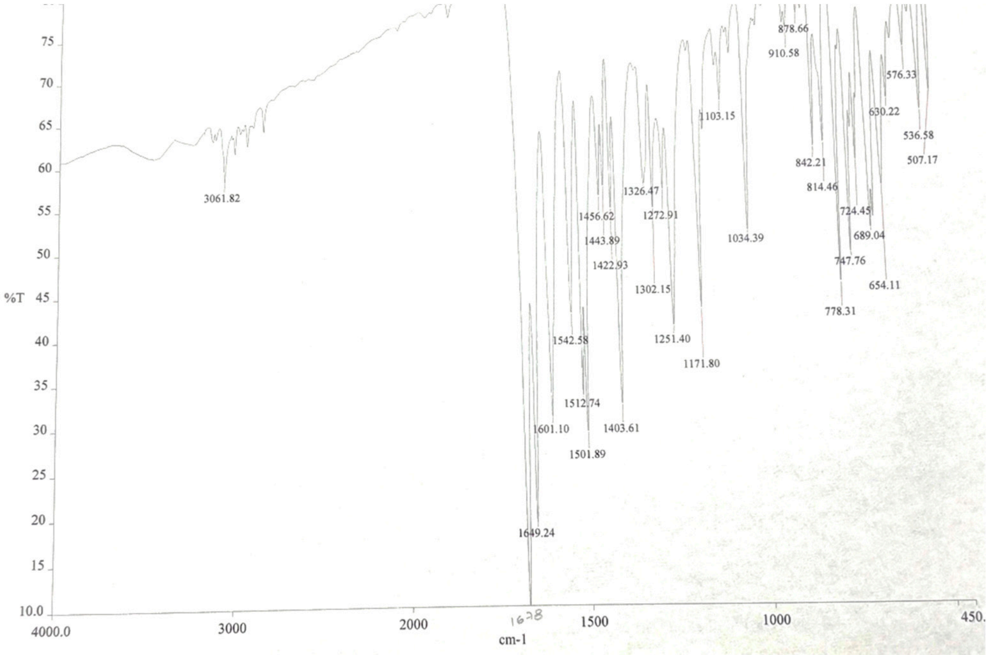

Spectrum S22: ^{1}H-NMR (DMSO-d$_6$). (*E*)-1-(6-chloro-4-(2-fluorophenyl)-2-methylquinolin-3-yl)-3-(4-metoxyphenyl)prop-2-en-1-one **(E007)**

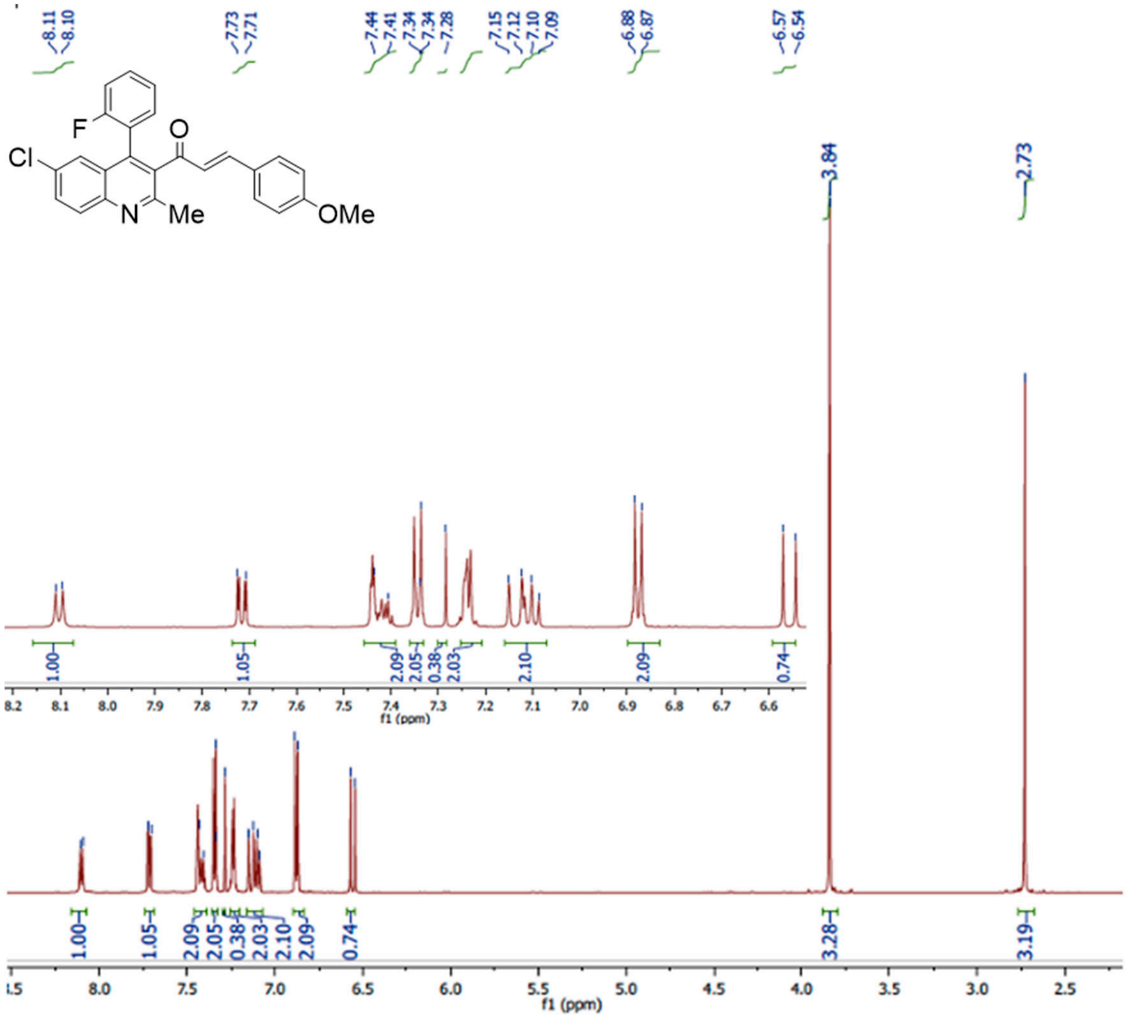

Spectrum S23: ^{13}C-NMR (DMSO-d_6). (*E*)-1-(6-chloro-4-(2-fluorophenyl)-2-methylquinolin-3-yl)-3-(4-metoxyphenyl)prop-2-en-1-one **(E007)**

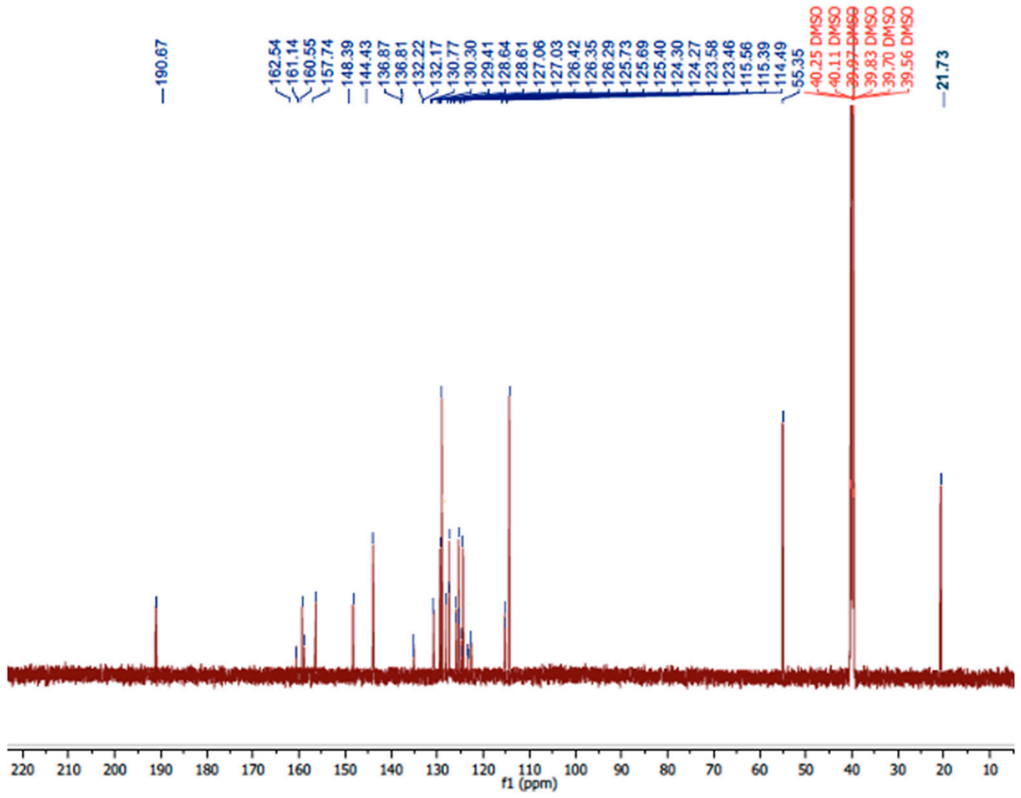

Spectrum S24: ^{13}C-NMR (DMSO-d_6). (*E*)-1-(6-chloro-4-(2-fluorophenyl)-2-methylquinolin-3-yl)-3-(4-metoxyphenyl)prop-2-en-1-one **(E007)**

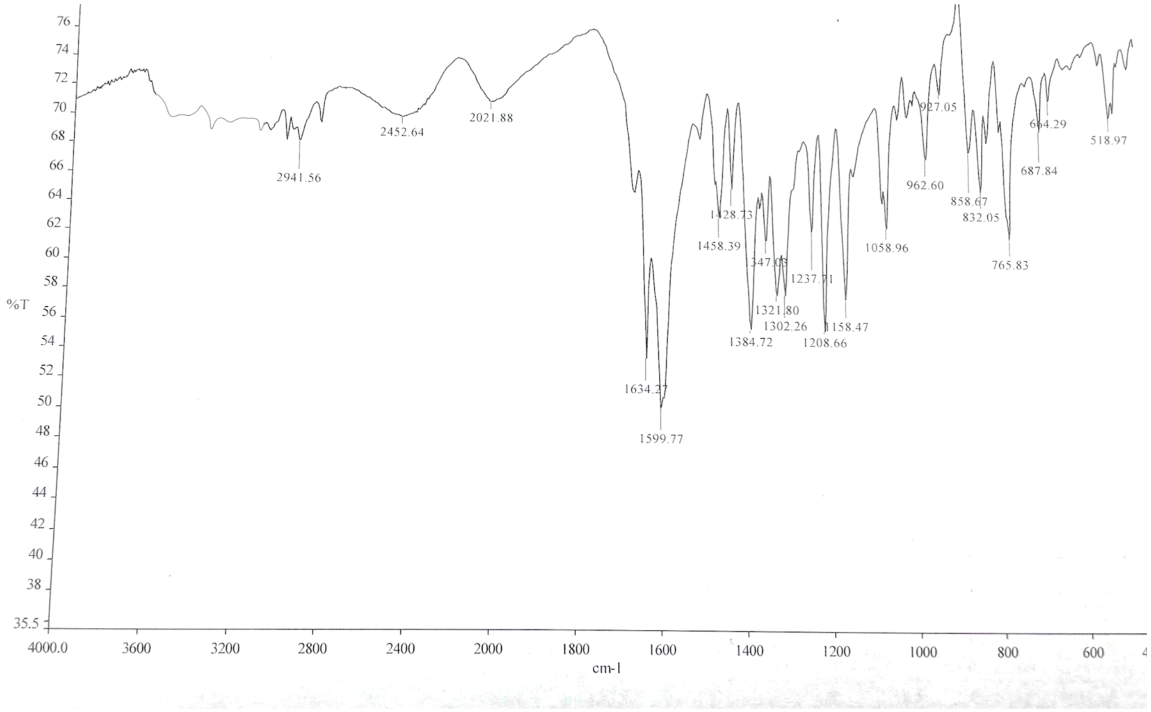

Spectrum S25: ^{1}H-NMR (DMSO-d_6). (*E*)-1-(6-chloro-4-(2-fluorophenyl)-2-methylquinolin-3-yl)-3-(6-metoxynapthalen-2-yl)prop-2-en-1-one **(E008)**

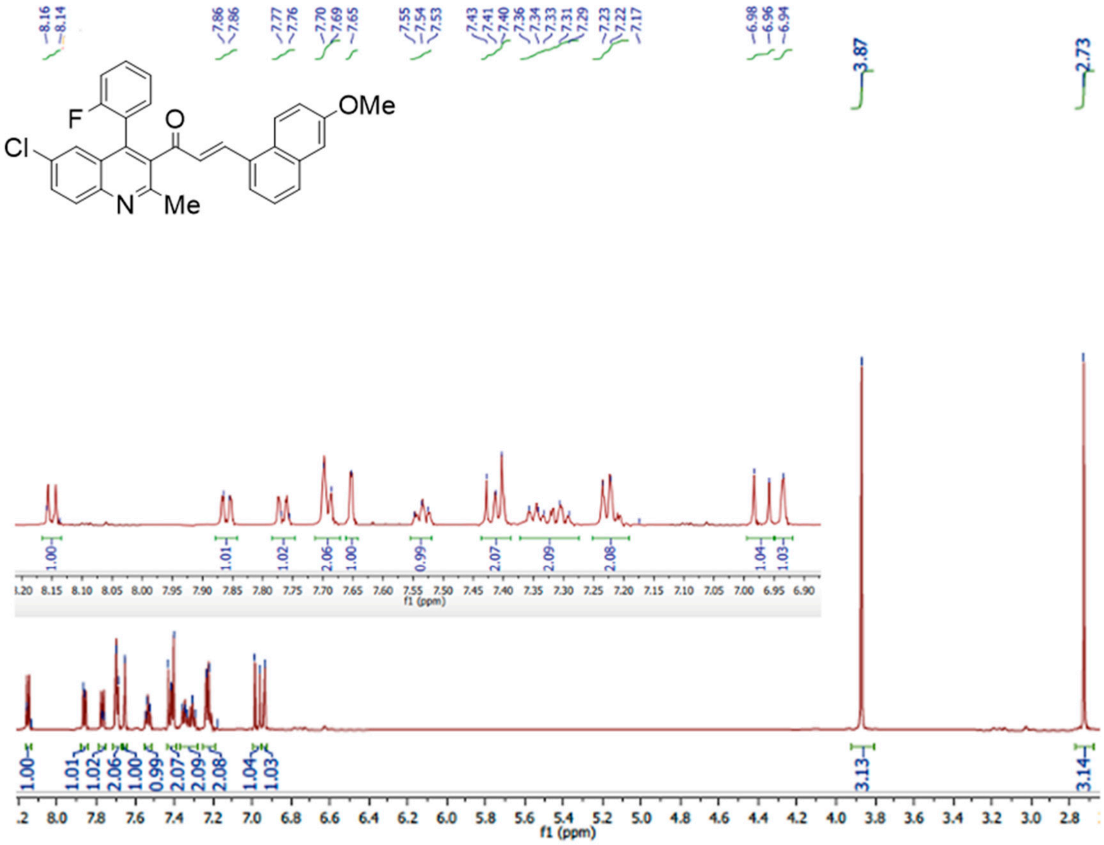

Spectrum S26: ^{13}C-NMR (DMSO-d_6). (*E*)-1-(6-chloro-4-(2-fluorophenyl)-2-methylquinolin-3-yl)-3-(6-metoxynapthalen-2-yl)prop-2-en-1-one **(E008)**

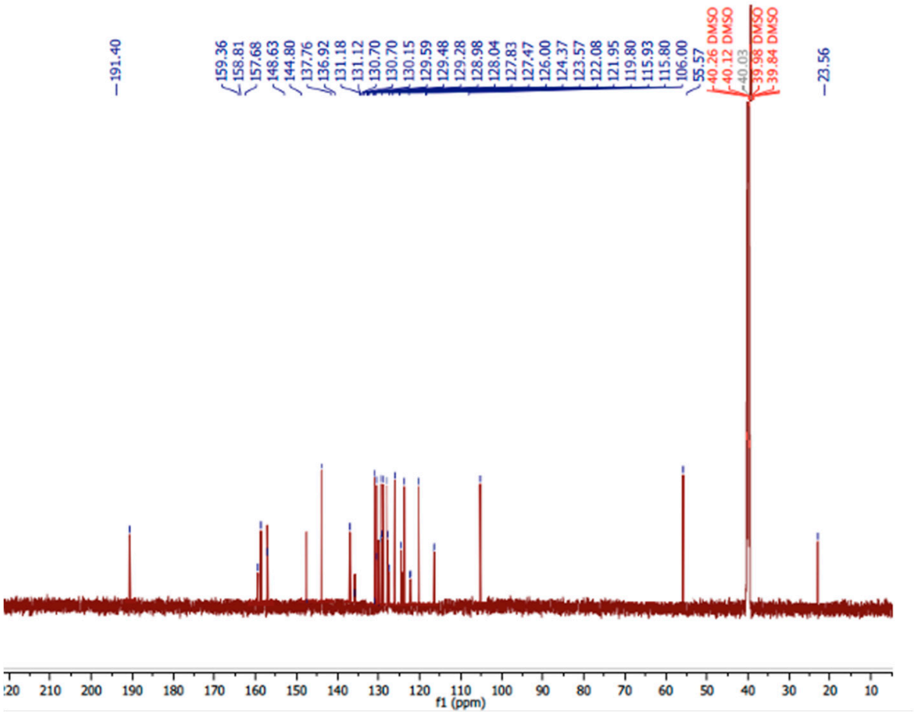

Spectrum S27: IR (KBr). (*E*)-1-(6-chloro-4-(2-fluorophenyl)-2-methylquinolin-3-yl)-3-(6-metoxynapthalen-2-yl)prop-2-en-1-one **(E008)**

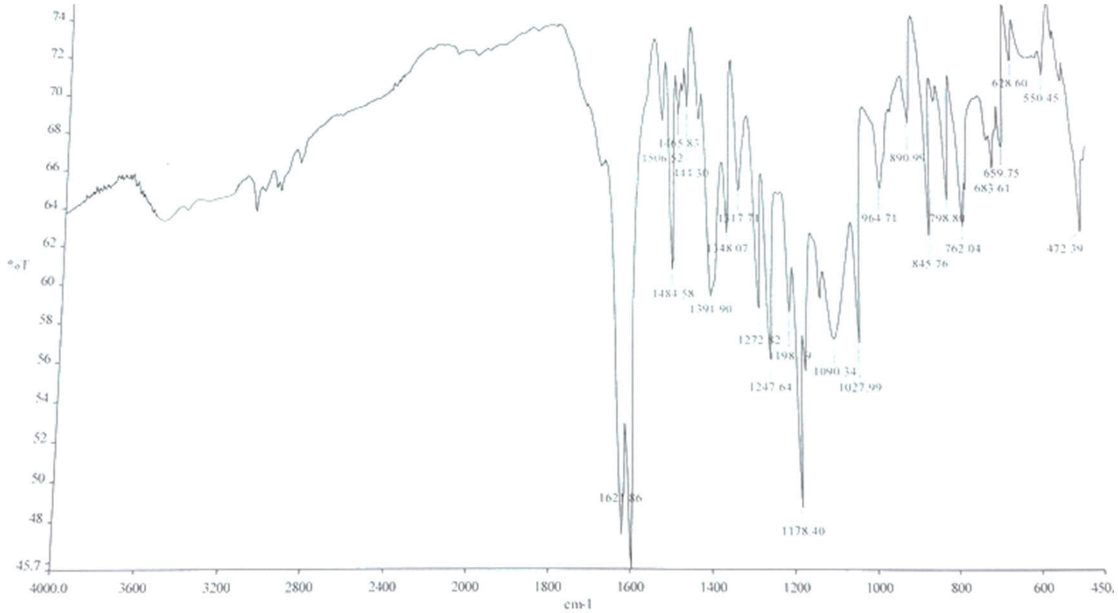

Spectrum S28: ^{1}H-NMR (DMSO-d$_6$). (*E*)-1-(6-chloro-4-(2-fluorophenyl)-2-methylquinolin-3-yl)-3-oxoprop-1en-1-yl)phenyl benzoate **(E009)**

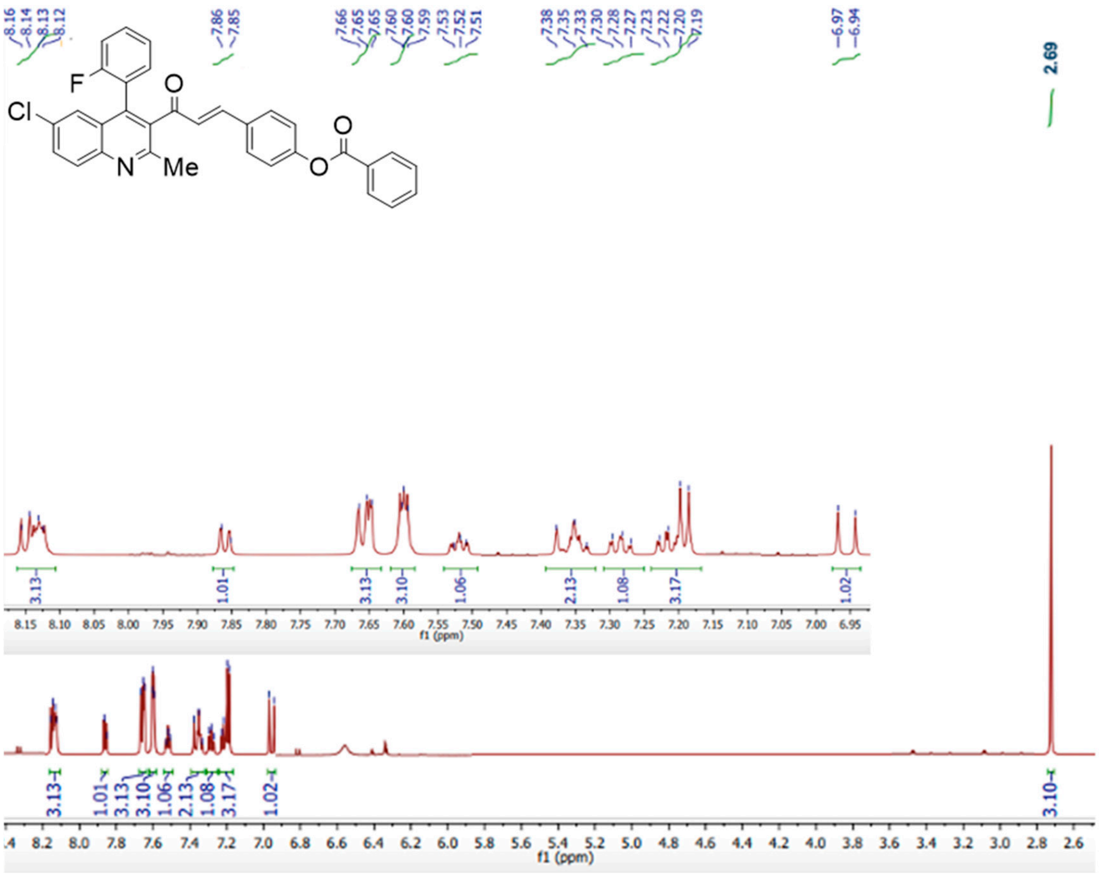

Spectrum S29: ^{13}C-NMR (DMSO-d_6). (*E*)-1-(6-chloro-4-(2-fluorophenyl)-2-methylquinolin-3-yl)-3-oxoprop-1en-1-yl)phenyl benzoate **(E009)**

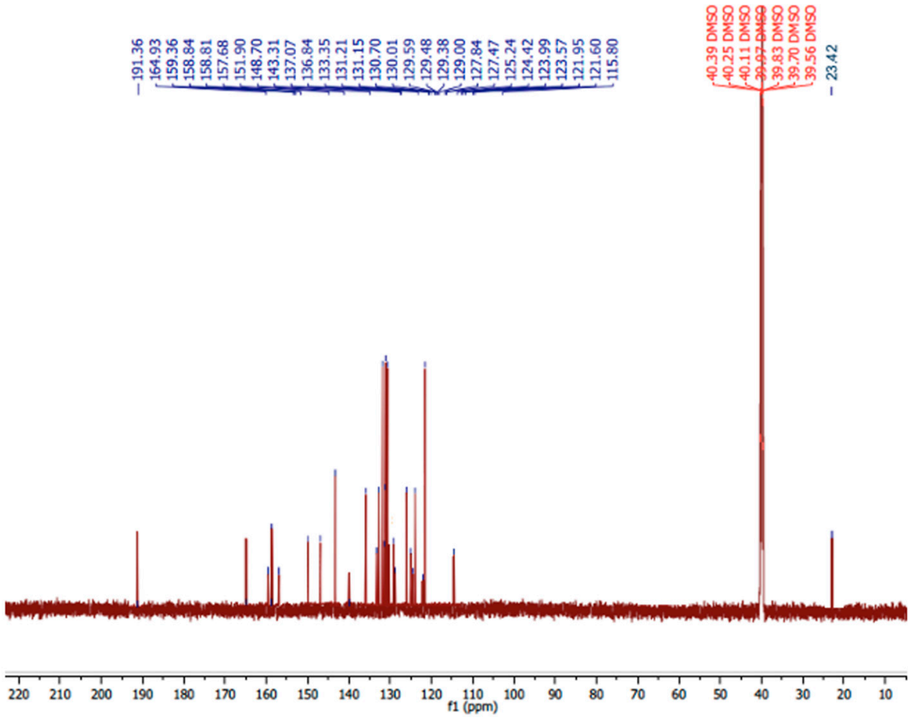

Spectrum S30: IR (KBr). (*E*)-1-(6-chloro-4-(2-fluorophenyl)-2-methylquinolin-3-yl)-3-oxoprop-1en-1-yl)phenyl benzoate **(E009)**

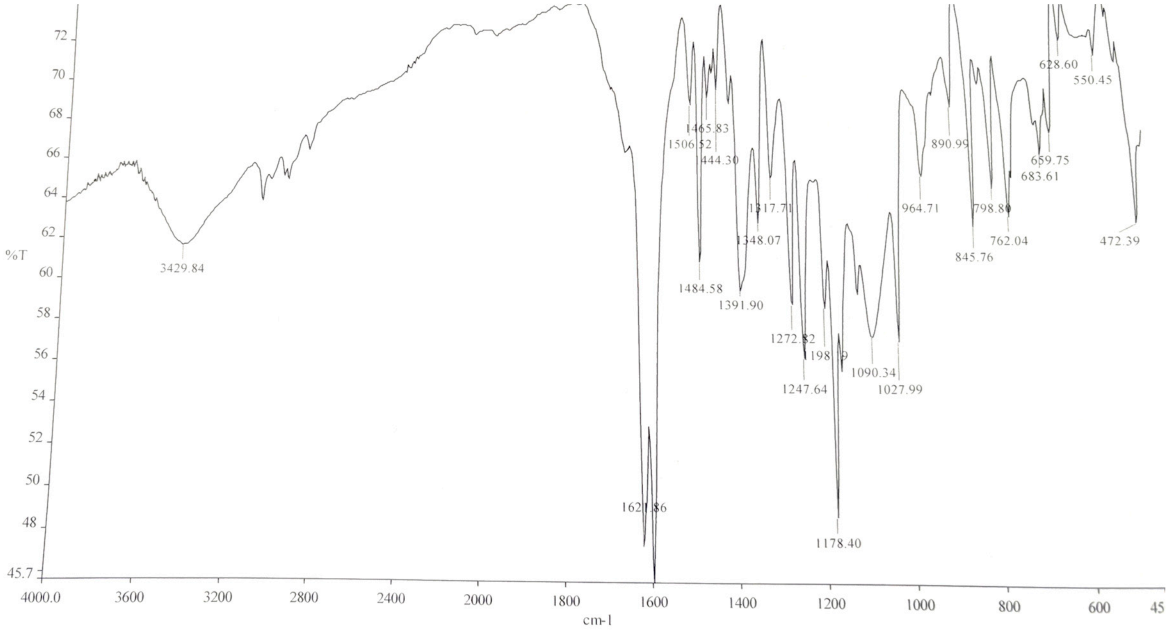

Spectrum S31: ^{1}H-NMR (DMSO-d$_6$). (*E*)-1-(6-chloro-4-(2-fluorophenyl)-2-methylquinolin-3-yl)-3- (4-cholrophenyl)prop-2-en-1-one **(E010).**

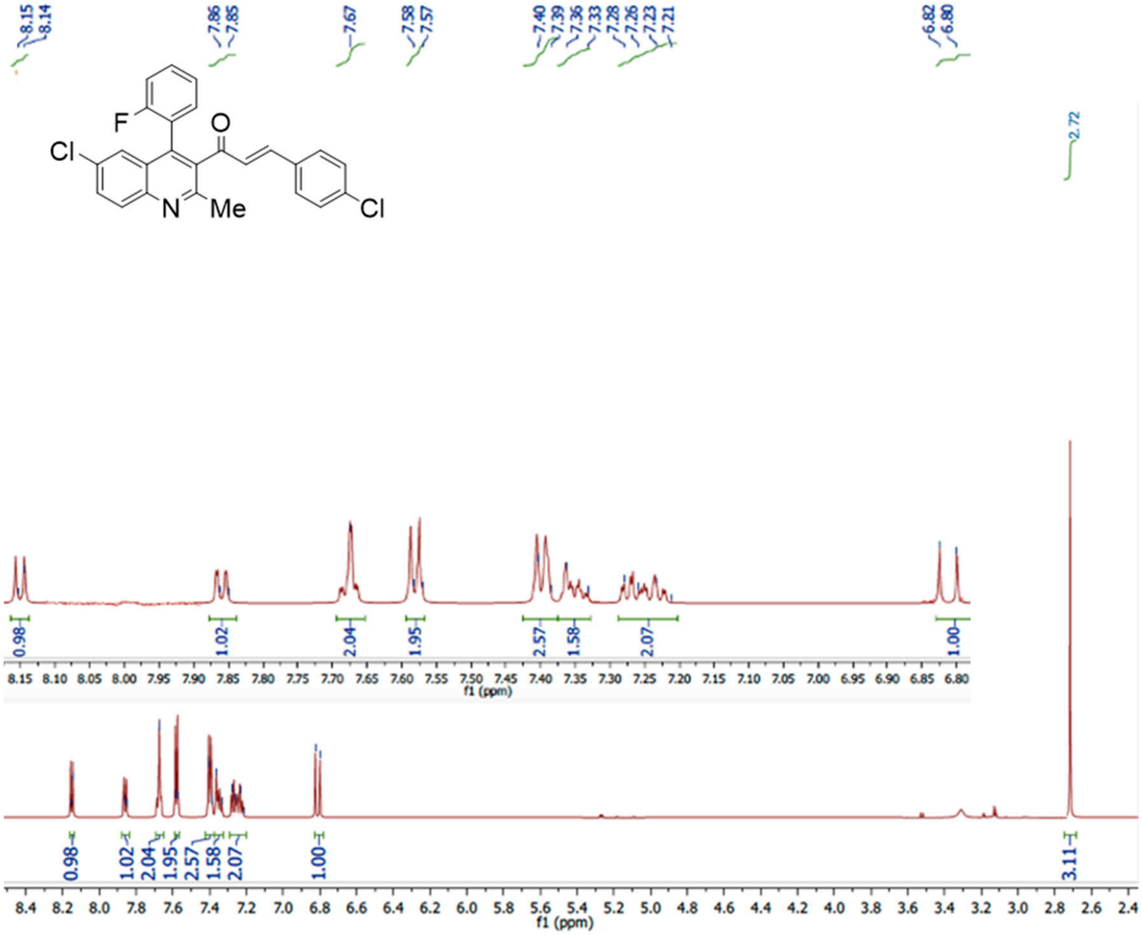

Spectrum S32: ^{13}C-NMR (DMSO-d$_6$). (*E*)-1-(6-chloro-4-(2-fluorophenyl)-2-methylquinolin-3-yl)-3- (4-cholrophenyl)prop-2-en-1-one **(E010).**

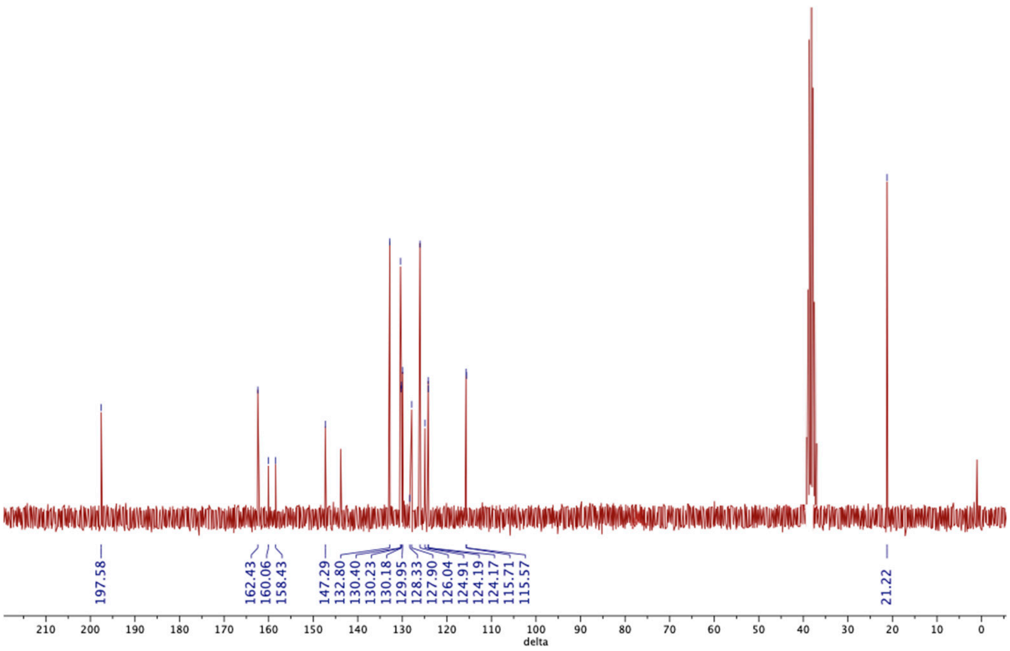

Spectrum S33: ^{13}C-NMR (DMSO-d$_6$). (*E*)-1-(6-chloro-4-(2-fluorophenyl)-2-methylquinolin-3-yl)-3- (4-cholrophenyl)prop-2-en-1-one **(E010).**

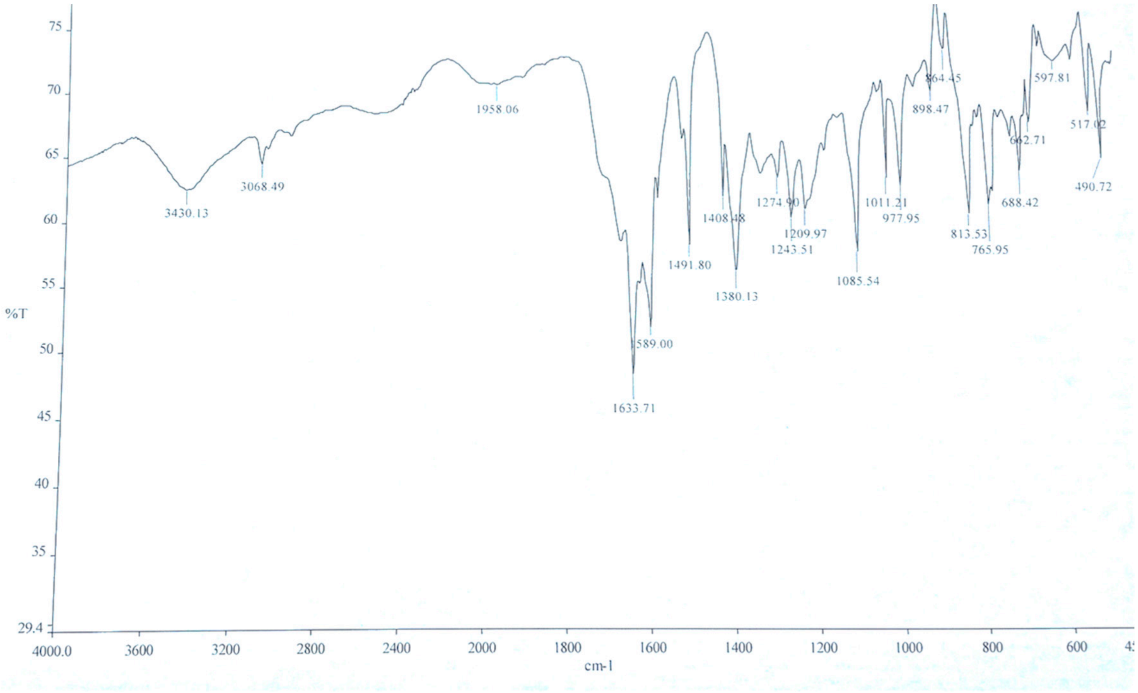

Spectrum S34: ^{1}H-NMR (DMSO-d_6). (*E*)-1-(6-chloro-4-(2-fluorophenyl)-2-methylquinolin-3-yl)-3-(3,5-difluorophenyl)prop-2-en-1-one **(E011)**

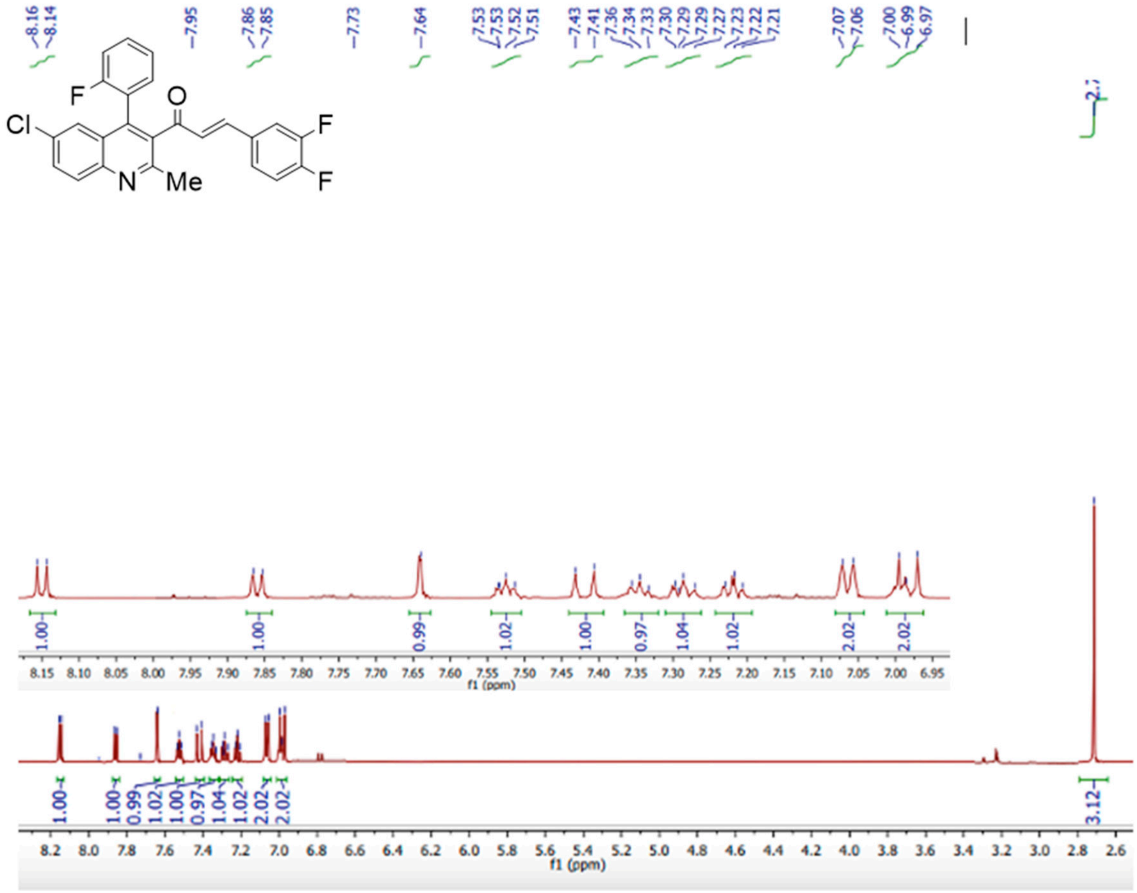

Spectrum S35: ^{13}C-NMR (DMSO-d_6). (*E*)-1-(6-chloro-4-(2-fluorophenyl)-2-methylquinolin-3-yl)-3-(3,5-difluorophenyl)prop-2-en-1-one **(E011)**

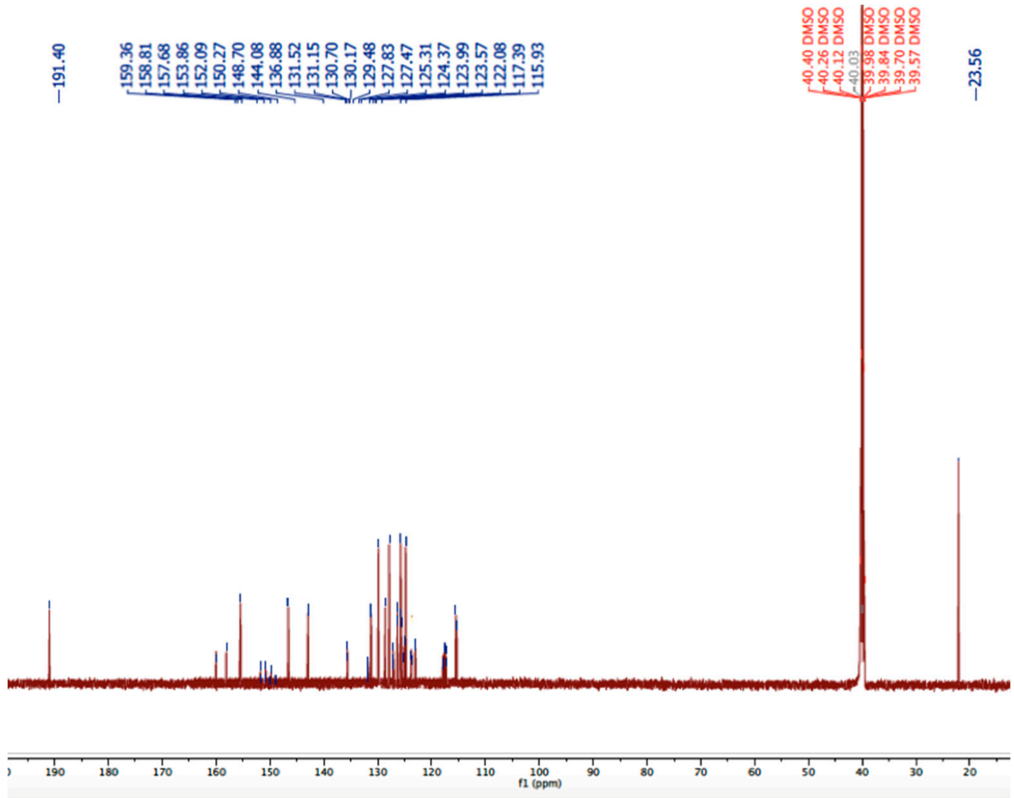

Spectrum S36: IR (KBr). (*E*)-1-(6-chloro-4-(2-fluorophenyl)-2-methylquinolin-3-yl)-3-(3,5-difluorophenyl)prop-2-en-1-one **(E011)**

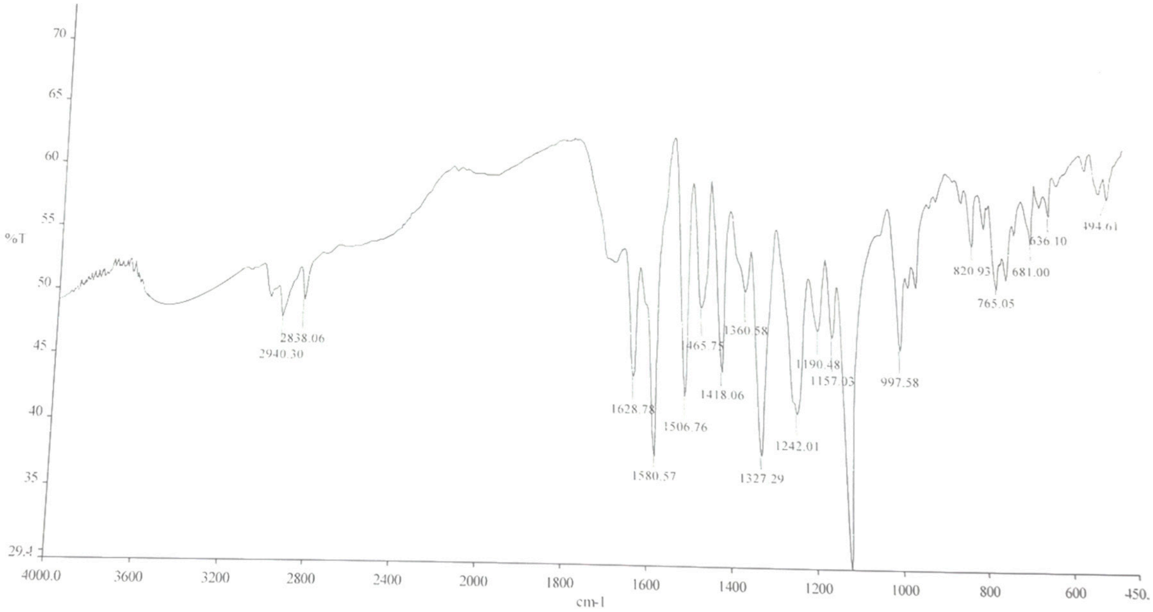

Spectrum S37: ^{1}H-NMR (DMSO-d$_6$). (*E*)-1-(6-chloro-4-(2-fluorophenyl)-2-methylquinolin-3-yl)-3-phenylprop-2-en-1-one **(E012)**

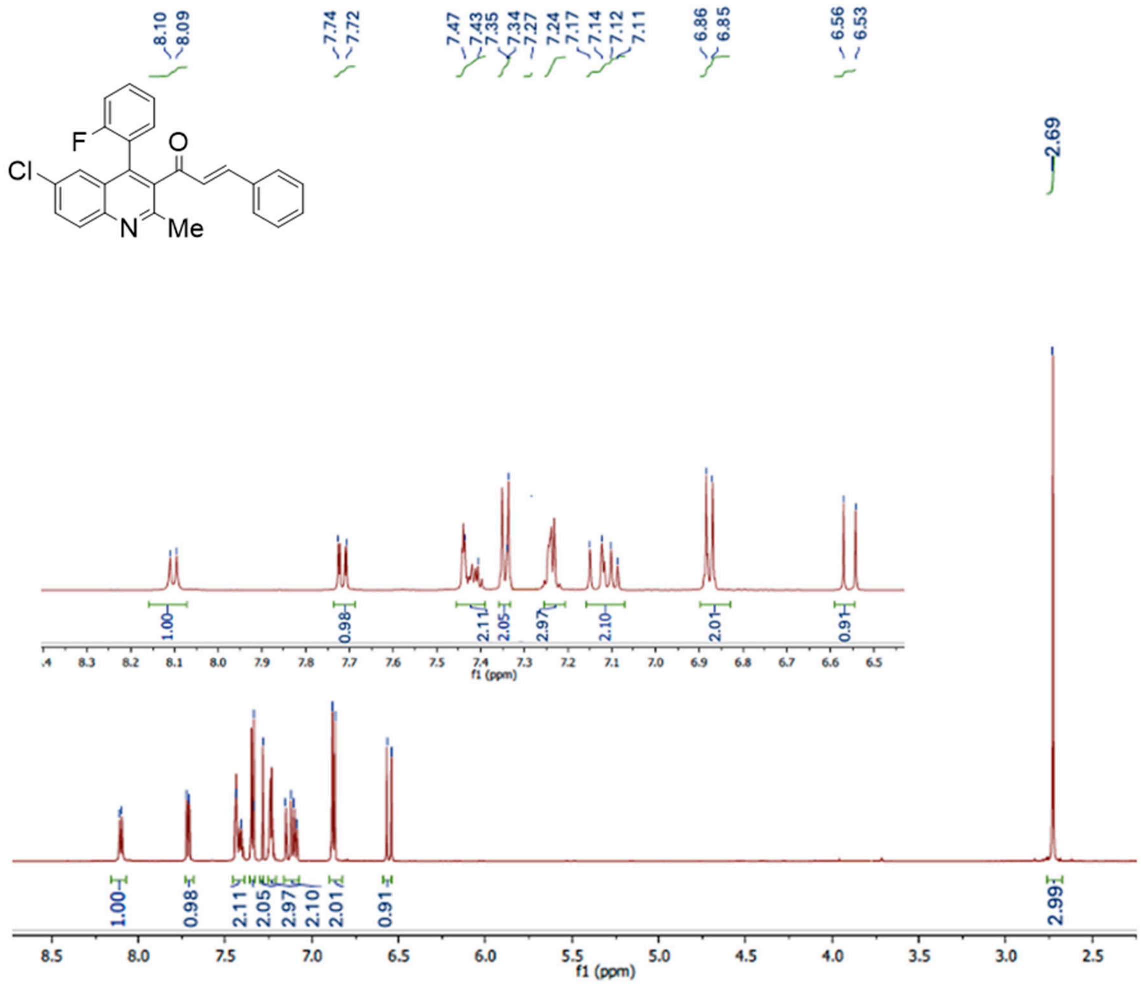

Spectrum S38: ^{13}C-NMR (DMSO-d_6). (*E*)-1-(6-chloro-4-(2-fluorophenyl)-2-methylquinolin-3-yl)-3-phenylprop-2-en-1-one **(E012)**

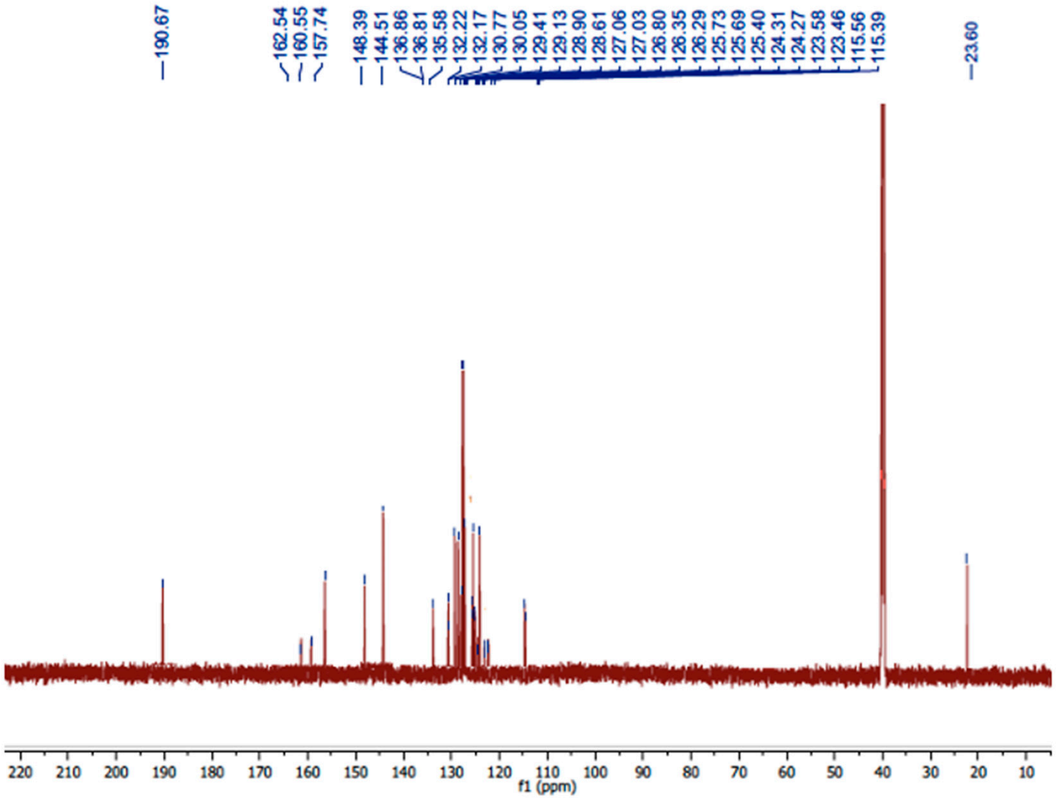

Spectrum S39: IR (KBr). (*E*)-1-(6-chloro-4-(2-fluorophenyl)-2-methylquinolin-3-yl)-3-phenylprop-2-en-1-one **(E012)**

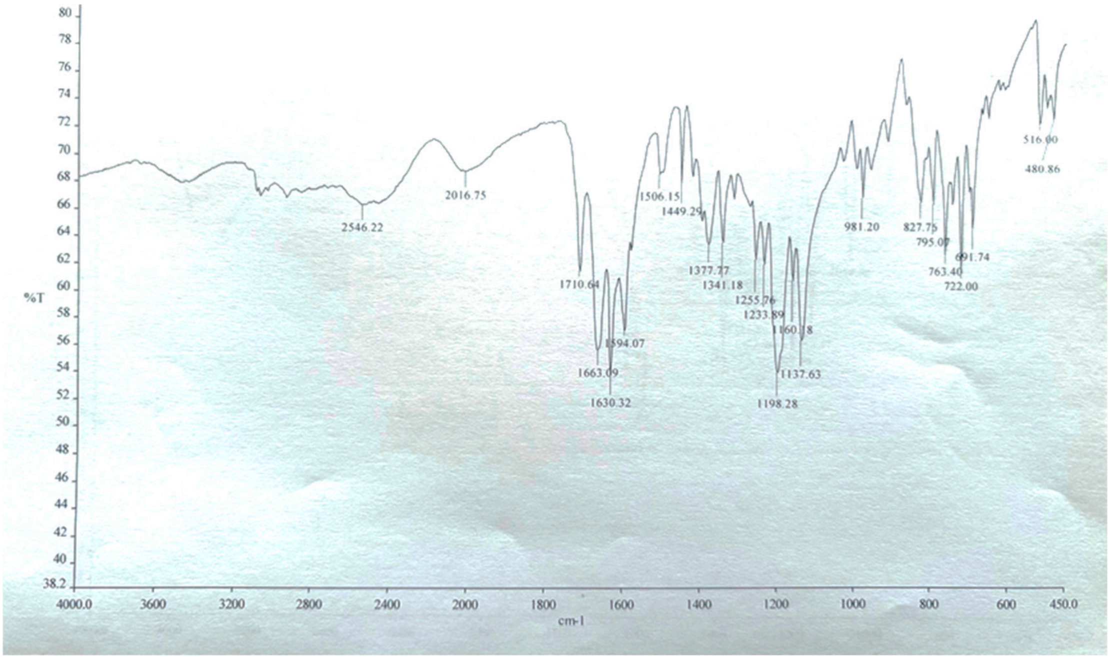

Supplement: Supplementary file 1 [file pharmaceuticals-18-01567-s001.zip › pharmaceuticals-3896206-supplementary.pdf]
